# Supplementary material for: Development of Semisynthetic Apoptosis-Inducing Agents Based on Natural Phenolic Acids Scaffold: Design, Synthesis and In-Vitro Biological Evaluation
Source: Molecules. 2022 Oct 9;27(19):6724. doi: 10.3390/molecules27196724 (PMC9571594; doi:10.3390/molecules27196724)
Supplement: Supplementary file 1 [file molecules-27-06724-s001.zip › Caffeic-13CNMR.pdf]

Shahera Ezzat_C_1

Microanalytical Unit - FOPCU - NMR laboratory
www.pharma.cu.edu.eg dir-mau.fopcu@pharma.cu.edu.eg

BRUKER

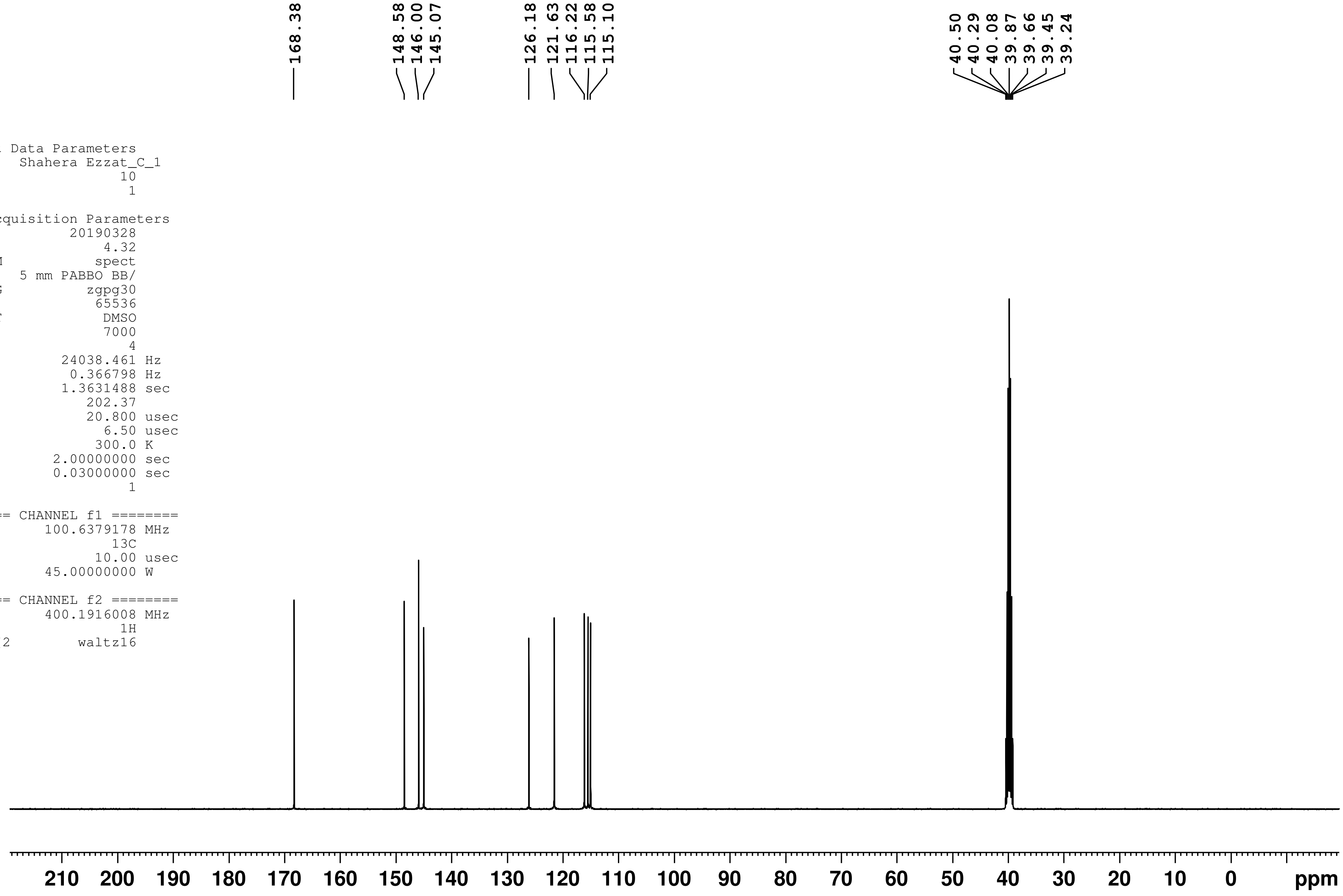


```
Current Data Parameters
NAME     Shahera Ezzat_C_1
EXPNO                10
PROCNO                1

F2 - Acquisition Parameters
Date_          20190328
Time               4.32
INSTRUM           spect
PROBHD   5 mm PABBO BB/
PULPROG         zgpg30
TD               65536
SOLVENT           DMSO
NS                7000
DS                   4
SWH          24038.461 Hz
FIDRES        0.366798 Hz
AQ           1.3631488 sec
RG              202.37
DW              20.800 usec
DE                6.50 usec
TE               300.0 K
D1          2.00000000 sec
D11         0.03000000 sec
TD0                  1

======== CHANNEL f1 ========
SFO1       100.6379178 MHz
NUC1               13C
P1               10.00 usec
PLW1       45.00000000 W

======== CHANNEL f2 ========
SFO2       400.1916008 MHz
NUC2                1H
CPDPRG[2       waltz16
```
