# Supplementary material for: Development of Semisynthetic Apoptosis-Inducing Agents Based on Natural Phenolic Acids Scaffold: Design, Synthesis and In-Vitro Biological Evaluation
Source: Molecules. 2022 Oct 9;27(19):6724. doi: 10.3390/molecules27196724 (PMC9571594; doi:10.3390/molecules27196724)
Supplement: Supplementary file 1 [file molecules-27-06724-s001.zip › Ferulic acid-NMR.pdf]

Shahera Ezzat_H_2

Microanalytical Unit - FOPCU - NMR laboratory
www.pharma.cu.edu.eg dir-mau.fopcu@pharma.cu.edu.eg

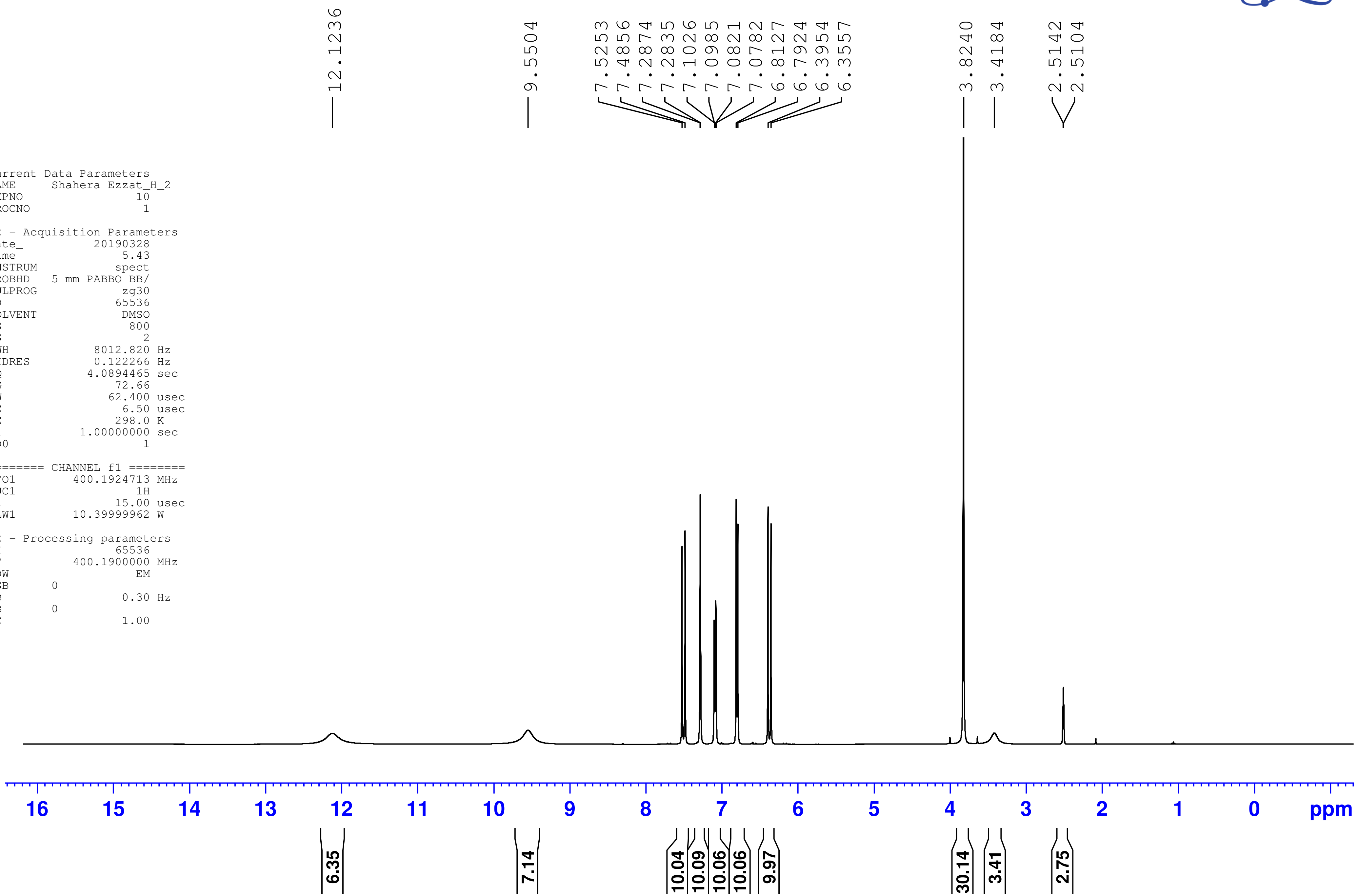


```
Current Data Parameters
NAME      Shahera Ezzat_H_2
EXPNO                   10
PROCNO                   1

F2 - Acquisition Parameters
Date_          20190328
Time               5.43
INSTRUM           spect
PROBHD   5 mm PABBO BB/
PULPROG            zg30
TD                65536
SOLVENT            DMSO
NS                  800
DS                    2
SWH            8012.820 Hz
FIDRES         0.122266 Hz
AQ            4.0894465 sec
RG                72.66
DW               62.400 usec
DE                 6.50 usec
TE                298.0 K
D1           1.00000000 sec
TD0                   1

======== CHANNEL f1 ========
SFO1        400.1924713 MHz
NUC1                 1H
P1                15.00 usec
PLW1        10.39999962 W

F2 - Processing parameters
SI                65536
SF          400.1900000 MHz
WDW                  EM
SSB       0
LB                 0.30 Hz
GB        0
PC                 1.00
```
