# Supplementary material for: Development of Semisynthetic Apoptosis-Inducing Agents Based on Natural Phenolic Acids Scaffold: Design, Synthesis and In-Vitro Biological Evaluation
Source: Molecules. 2022 Oct 9;27(19):6724. doi: 10.3390/molecules27196724 (PMC9571594; doi:10.3390/molecules27196724)
Supplement: Supplementary file 1 [file molecules-27-06724-s001.zip › HGNC ID.pdf]

Table: HGNC ID of the used genes

Gene	HGNC ID
P53	**11998**
CASP-3	**1504**
Bax	**959**
MMP-2	**7166**
MMP-9	**7176**
