# Supplementary material for: Development of Semisynthetic Apoptosis-Inducing Agents Based on Natural Phenolic Acids Scaffold: Design, Synthesis and In-Vitro Biological Evaluation
Source: Molecules. 2022 Oct 9;27(19):6724. doi: 10.3390/molecules27196724 (PMC9571594; doi:10.3390/molecules27196724)
Supplement: Supplementary file 1 [file molecules-27-06724-s001.zip › NMR of compound17.pdf]

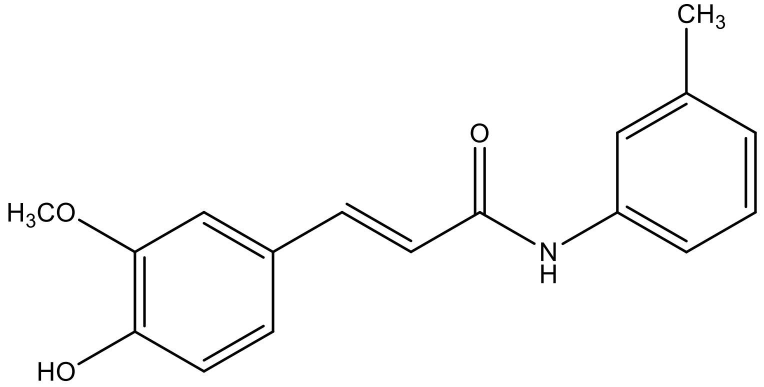

SpinWorks 2.5: Omar Adel_H_FA8

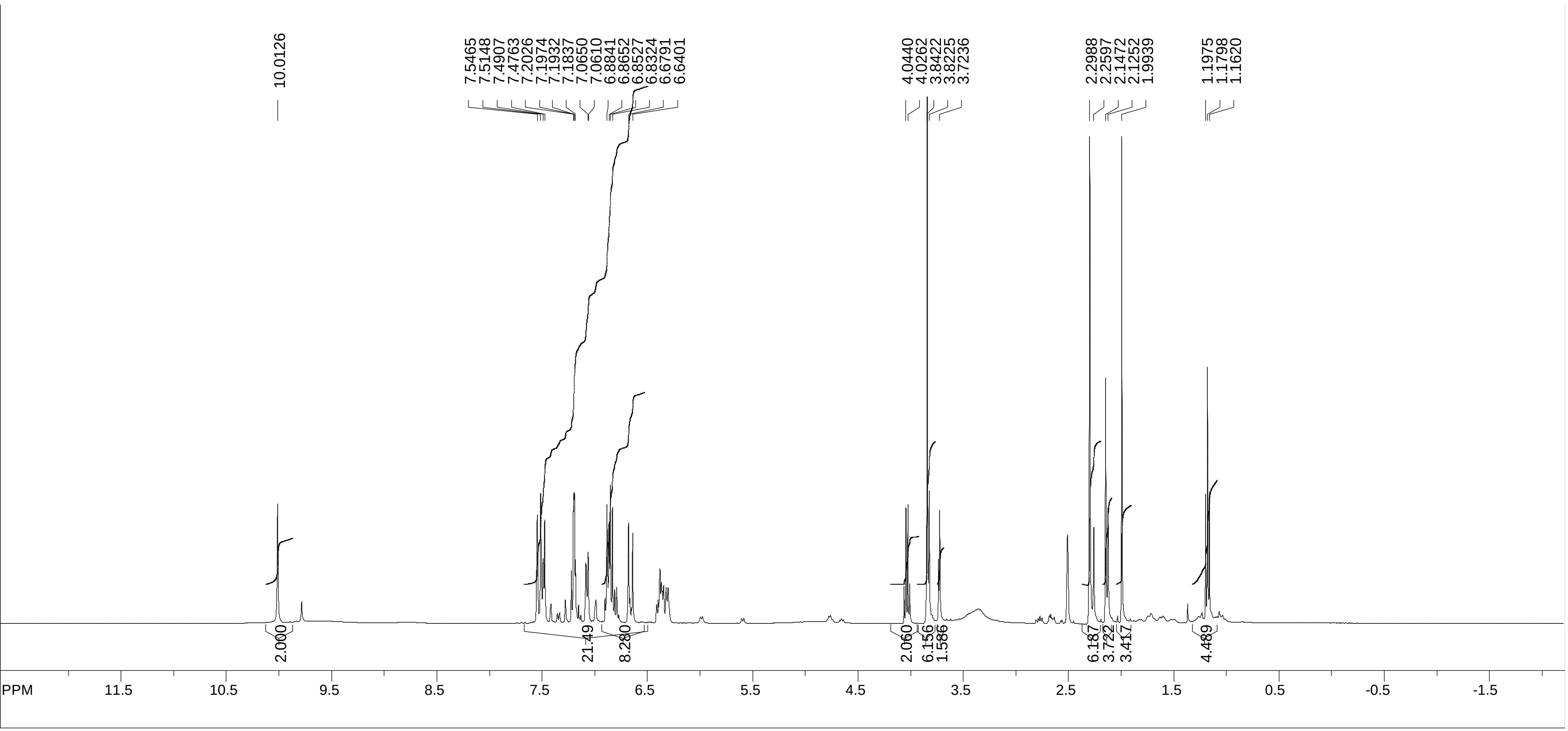

file: D:\MSA\New folder\Omar Adel_H_FA8\10\fid expt: <zg30>
transmitter freq.: 400.192471 MHz
time domain size: 65536 points
width: 8012.82 Hz = 20.022417 ppm = 0.122266 Hz/pt
number of scans: 32

freq. of 0 ppm: 400.190000 MHz
processed size: 65536 complex points
LB: 0.300 GB: 0.0000

SpinWorks 2.5: Omar Adel_H_FA8

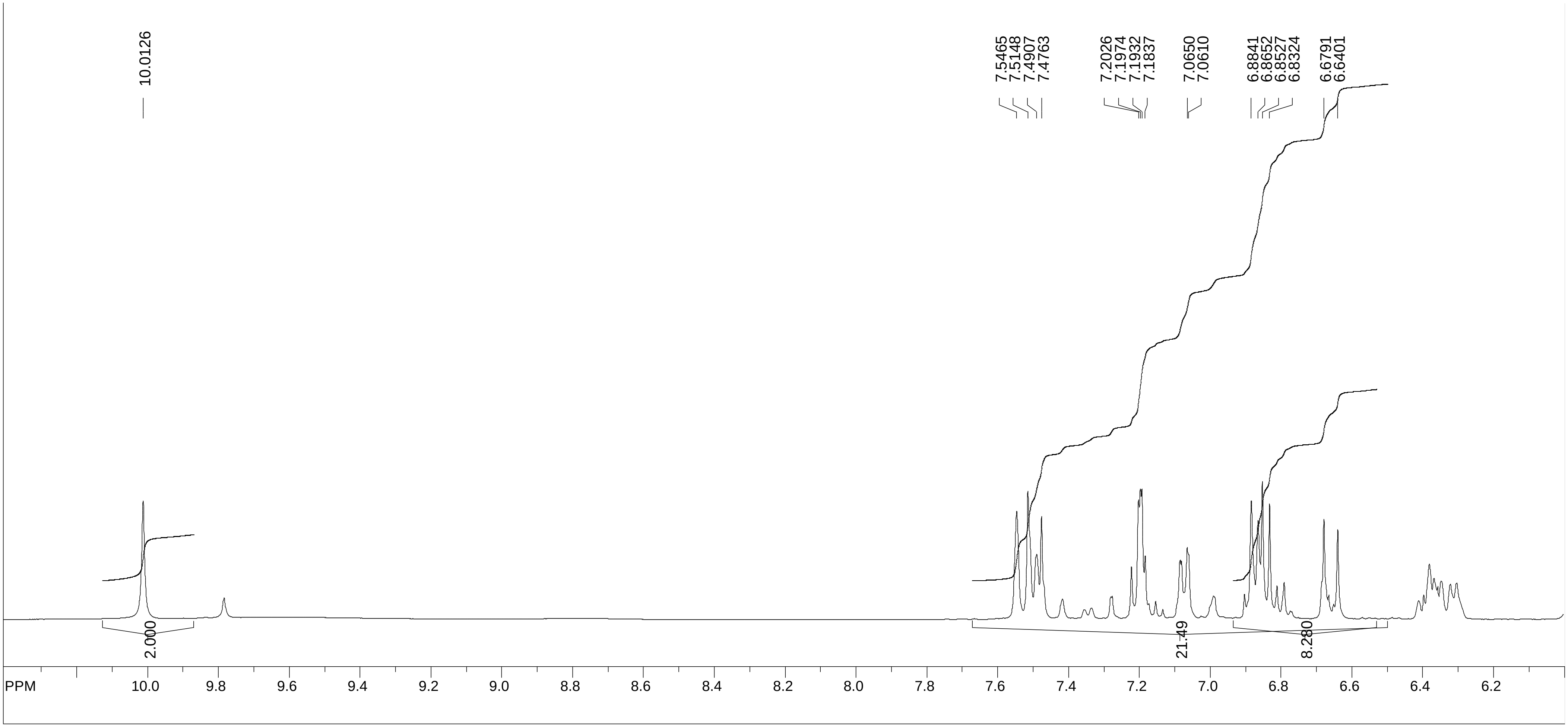

file: D:\MSA\New folder\Omar Adel_H_FA8\10\fid   expt: <zg30>
transmitter freq.: 400.192471 MHz
time domain size: 65536 points
width: 8012.82 Hz = 20.022417 ppm = 0.122266 Hz/pt
number of scans: 32

freq. of 0 ppm: 400.190000 MHz
processed size: 65536 complex points
LB: 0.300   GB: 0.0000

SpinWorks 2.5: Omar Adel_H_FA8

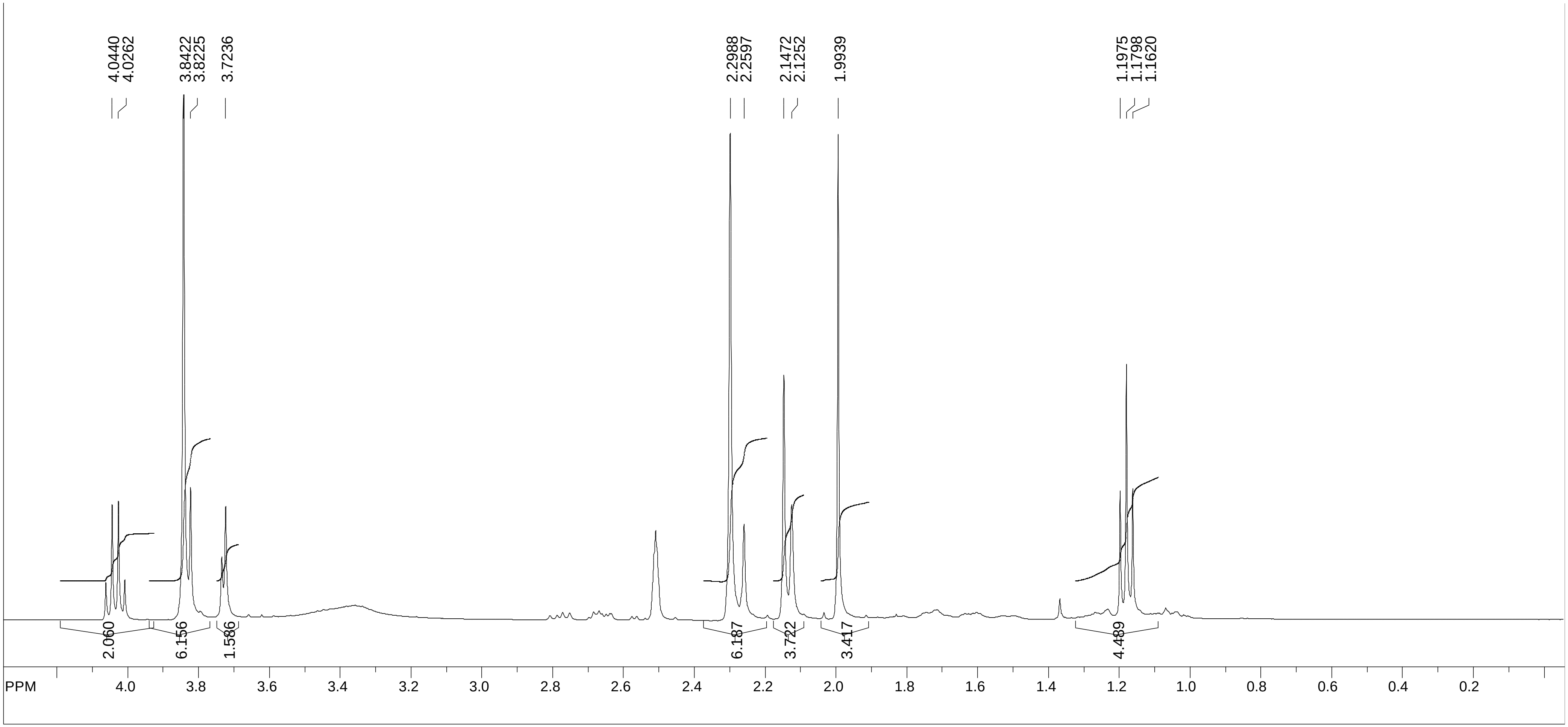

file: D:\MSA\New folder\Omar Adel_H_FA8\10\fid   expt: <zg30>
transmitter freq.: 400.192471 MHz
time domain size: 65536 points
width: 8012.82 Hz = 20.022417 ppm = 0.122266 Hz/pt
number of scans: 32

freq. of 0 ppm: 400.190000 MHz
processed size: 65536 complex points
LB: 0.300   GB: 0.0000
