# Supplementary material for: Development of Semisynthetic Apoptosis-Inducing Agents Based on Natural Phenolic Acids Scaffold: Design, Synthesis and In-Vitro Biological Evaluation
Source: Molecules. 2022 Oct 9;27(19):6724. doi: 10.3390/molecules27196724 (PMC9571594; doi:10.3390/molecules27196724)
Supplement: Supplementary file 1 [file molecules-27-06724-s001.zip › Report CCA.pdf]

Researcher : DR Moh.Abdelbasir email: m_a_baser@yahoo.com *mob.01*
Assay : Cell Cycle Analysis
Samples : 12 Samples
Cell lines : **Caco2**
Ref. : ---
Date : 25-06-2019
Reader : ***BD FACSCalibur***
Kit used : ab139418_Propidium Iodide Flow Cytometry Kit/BD
Solvent : **---**
Assay samples :Cell culture

Key for sample codes

Cpd. No.	Cpd. No.	Cpd. No.	Cpd. No.
**CA1**	**3**	FA1	**12**
**CA2**	**4**	FA2	**13**
**CA3**	**5**	FA3	**14**
**CA5**	**6**	FA5	**15**
**CA6**	**7**	FA7	**16**
**CA7**	**8**	FA8	**17**
**CA9**	**9**	FA9	**18**
**CA11**	**10**	FA11	**19**
**CA12**	**11**	FA12	**20**

Report

Sample data			Results				
ser	code	Used conc. uM	%G0-G1	%S	%G2-M	%Pre G1	Comment
1	s1/Caco2		46.29 ±3.1	33.22 ±1.99	20.49± 1.92	11.31 ±0.81	PreG1 apoptosis&Cell growth arrest@G2/M
2	s2/Caco2		39.77 ±2.29	34.08 ±2.63	26.15± 3.26	13.22 ±0.63	
3	CA3/Caco2		41.52 ±2.61	31.62 ±1.79	26.86± 2.75	7.56 ±0.39	
4	CA6/Caco2		28.68 ±1.64	26.41 ±2.15	44.91± 3.42	18.26 ±1.42	
5	CA9/Caco2		38.51 ±1.95	31.44 ±2.87	30.05± 2.59	9.62 ±0.37	
6	CA11/Caco2		29.75 1.73±	28.27 ±1.99	41.98± 2.18	23.42 ±2.12	
7	CA12/Caco2		41.09 ±2.76	33.36 ±2.73	25.55± 2.47	14.04 ±0.95	
8	FA3/Caco2		34.28 ±1.74	31.15 ±3.17	34.57± 4.16	16.51 ±0.63	
9	FA5/Caco2		26.59 ±2.67	22.58 ±2.91	50.83± 3.49	25.17 ±3.29	
10	FA8/Caco2		42.11 ±3.41	29.43 ±3.16	28.46± 2.64	12.11 ±0.97	
11	FA9/Caco2		33.26 ±4.29	29.12 ±4.05	37.62± 2.47	12.92 ±2.16	
12	FA12/Caco2		39.12 ±2.58	34.04 ±2.69	26.84± 2.34	8.62 ±0.69	
***	cont.Caco2		53.61 ±3.16	37.95 ±2.25	8.44± 0.63	1.78 ±0.08	

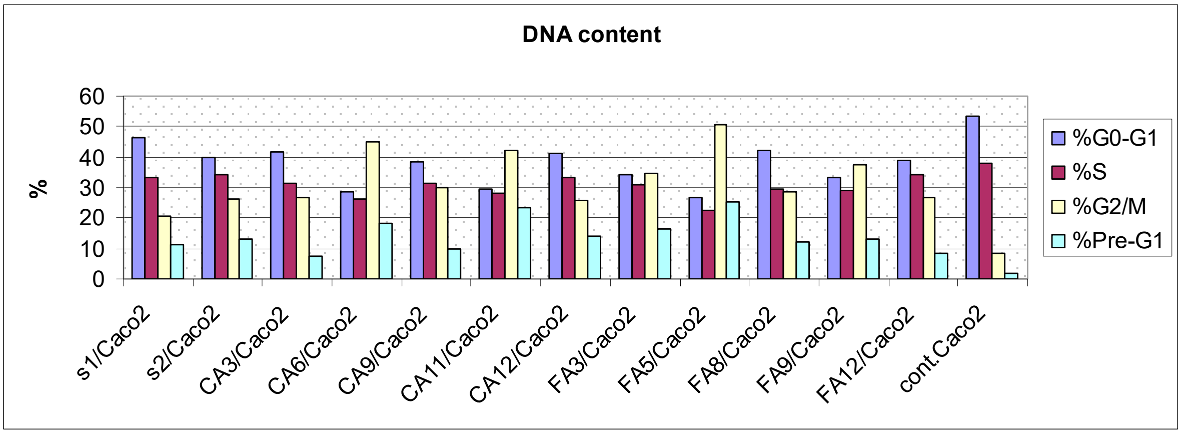

Detailed results

s		Apoptosis			Necrosis
		Total	Early	Late	
1	s1/Caco2	11.31	3.41	5.98	1.92
2	s2/Caco2	13.22	5.17	6.48	1.57
3	CA3/Caco2	7.56	2.59	3.93	1.04
4	CA6/Caco2	18.26	4.75	11.37	2.14
5	CA9/Caco2	9.62	3.72	4.54	1.36
6	CA11/Caco2	23.42	7.59	12.69	3.14
7	CA12/Caco2	14.04	2.47	9.5	2.07
8	FA3/Caco2	16.51	3.99	10.26	2.26
9	FA5/Caco2	25.17	5.79	16.57	2.81
10	FA8/Caco2	12.11	4.25	6.25	1.61
11	FA9/Caco2	12.92	3.88	7.31	1.73
12	FA12/Caco2	8.62	2.19	5.19	1.24
***	cont.Caco2	1.78	0.89	0.25	0.64

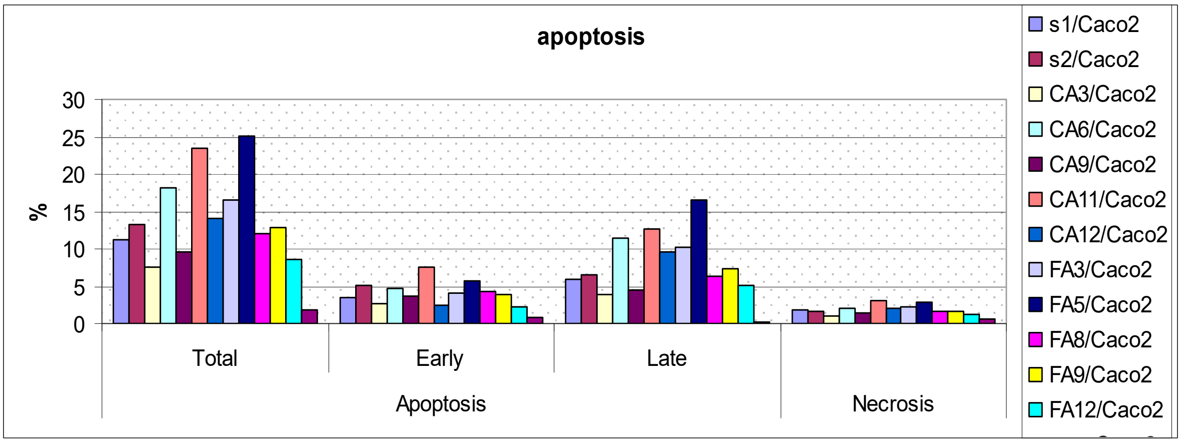

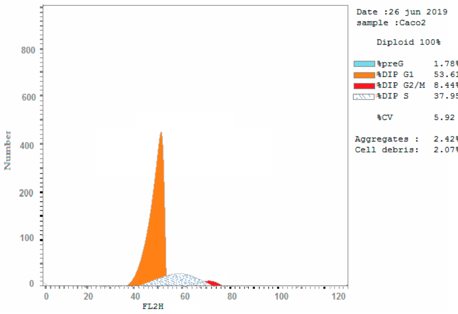
Date :26 jun 2019
sample :Caco2
Diploid 100%
%preG 1.78%
%DIP G1 53.61%
%DIP G2/M 8.44%
%DIP S 37.95%
%CV 5.92
Aggregates : 2.42%
Cell debris: 2.07%
Number
FL2H

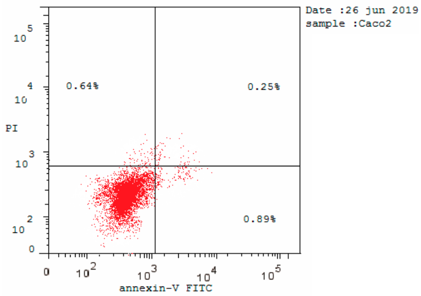
Date :26 jun 2019
sample :Caco2
0.64%
0.25%
0.89%
PI
annexin-V FITC

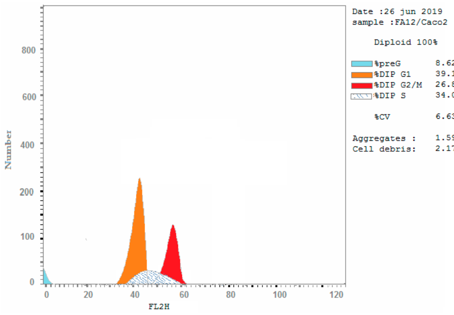
Date :26 jun 2019
sample :FA12/Caco2
Diploid 100%
%preG 8.62%
%DIP G1 39.12%
%DIP G2/M 26.84%
%DIP S 34.04%
%CV 6.63
Aggregates : 1.59%
Cell debris: 2.17%
Number
FL2H

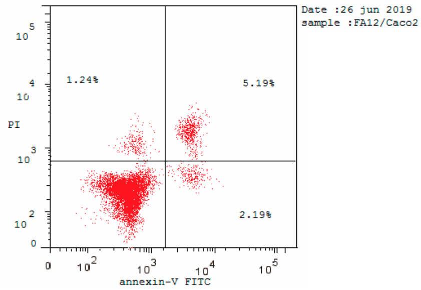
Date :26 jun 2019
sample :FA12/Caco2
1.24%
5.19%
2.19%
PI
annexin-V FITC

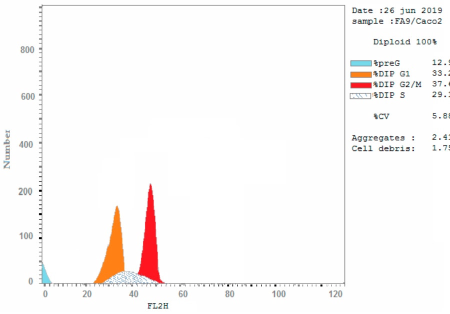
Date :26 jun 2019
sample :FA9/Caco2
Diploid 100%
%preG 12.92%
%DIP G1 33.26%
%DIP G2/M 37.62%
%DIP S 29.12%
%CV 5.88
Aggregates : 2.41%
Cell debris: 1.75%
Number
FL2H

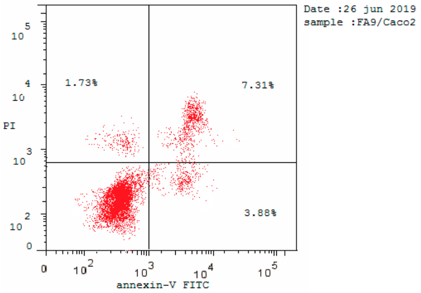
Date :26 jun 2019
sample :FA9/Caco2
1.73%
7.31%
3.88%
PI
annexin-V FITC

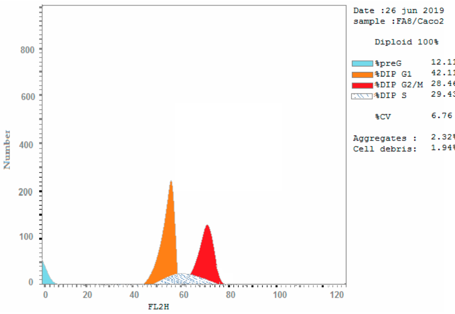
Date :26 jun 2019
sample :FA8/Caco2
Diploid 100%
%preG 12.11%
%DIP G1 42.11%
%DIP G2/M 28.46%
%DIP S 29.43%
%CV 6.76
Aggregates : 2.32%
Cell debris: 1.94%
Number
FL2H

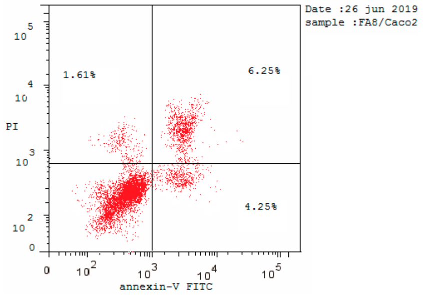
Date :26 jun 2019
sample :FA8/Caco2
1.61%
6.25%
4.25%
PI
annexin-V FITC

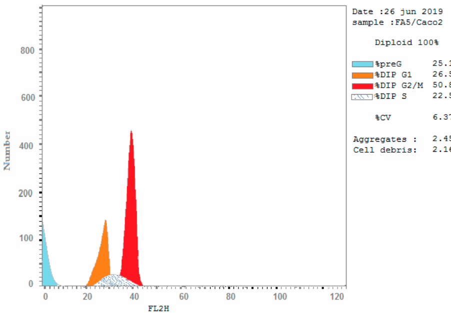
Date :26 jun 2019
sample :FA5/Caco2
Diploid 100%
%preG 25.17%
%DIP G1 26.59%
%DIP G2/M 50.83%
%DIP S 22.58%
%CV 6.37
Aggregates : 2.45%
Cell debris: 2.16%
Number
FL2H

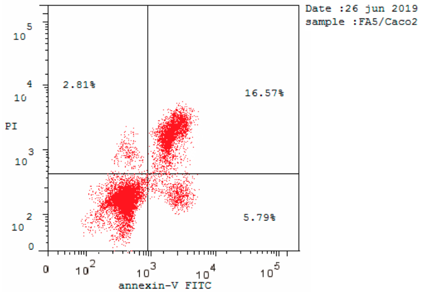
Date :26 jun 2019
sample :FA5/Caco2
2.81%
16.57%
5.79%
PI
annexin-V FITC

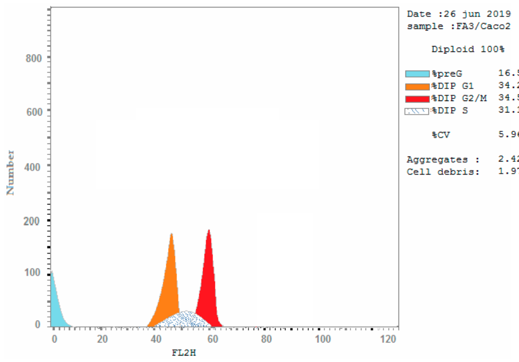
Date :26 jun 2019
sample :FA3/Caco2
Diploid 100%
%preG 16.51%
%DIP G1 34.28%
%DIP G2/M 34.57%
%DIP S 31.15%
%CV 5.96
Aggregates : 2.42%
Cell debris: 1.97%
Number
FL2H

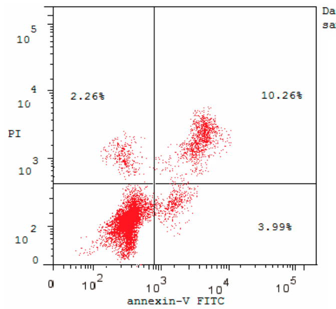
Date :26 jun 2019
sample :FA3/Caco2
2.26%
10.26%
3.99%
PI
annexin-V FITC

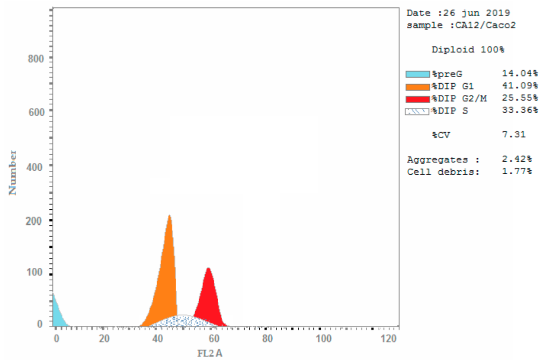
Date :26 jun 2019
sample :CA12/Caco2
Diploid 100%
%preG 14.04%
%DIP G1 41.09%
%DIP G2/M 25.55%
%DIP S 33.36%
%CV 7.31
Aggregates : 2.42%
Cell debris: 1.77%
Number
FL2 A

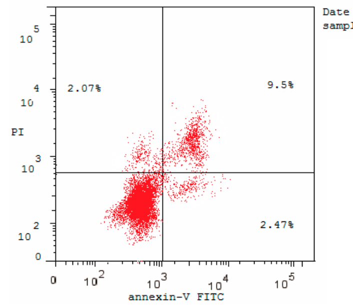
Date :26 jun 2019
sample :CA12/Caco2
2.07%
9.5%
2.47%
PI
annexin-V FITC

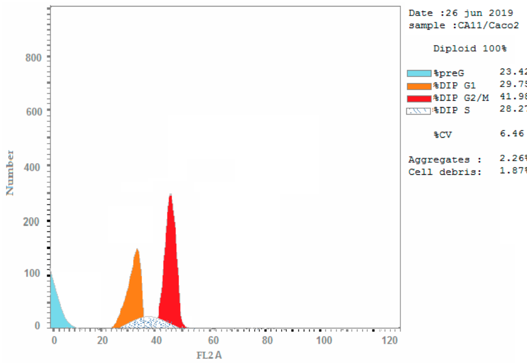
Date :26 jun 2019
sample :CA11/Caco2
Diploid 100%
%preG 23.42%
%DIP G1 29.75%
%DIP G2/M 41.98%
%DIP S 28.27%
%CV 6.46
Aggregates : 2.26%
Cell debris: 1.87%
Number
FL2 A

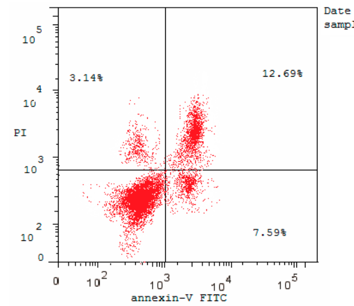
Date :26 jun 2019
sample :CA11/Caco2
3.14%
12.69%
7.59%
PI
annexin-V FITC

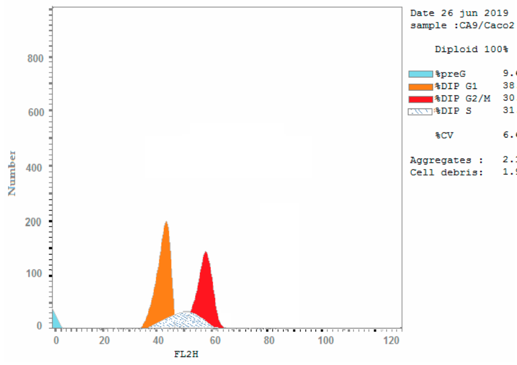
Date 26 jun 2019
sample :CA9/Caco2
Diploid 100%
%preG 9.62%
%DIP G1 38.51%
%DIP G2/M 30.05%
%DIP S 31.44%
%CV 6.67
Aggregates : 2.11%
Cell debris: 1.93%
Number
FL2H

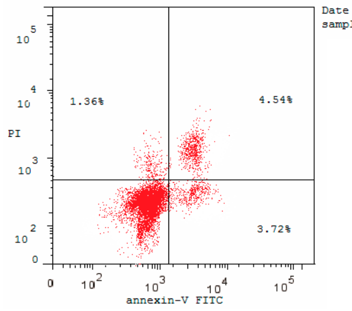
Date 26 jun 2019
sample :CA9/Caco2
1.36%
4.54%
3.72%
PI
annexin-V FITC

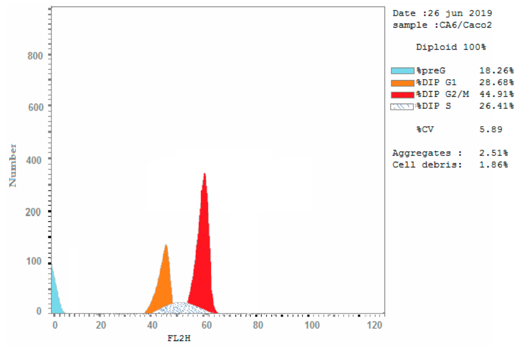
Date :26 jun 2019
sample :CA6/Caco2
Diploid 100%
%preG 18.26%
%DIP G1 28.68%
%DIP G2/M 44.91%
%DIP S 26.41%
%CV 5.89
Aggregates : 2.51%
Cell debris: 1.86%
Number
FL2H

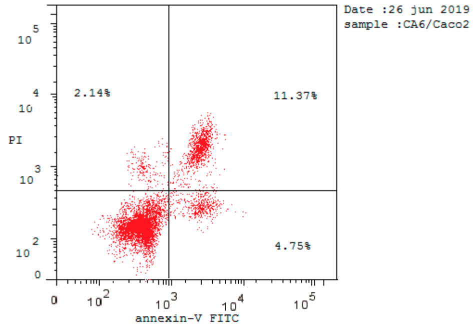
Date :26 jun 2019
sample :CA6/Caco2
2.14%
11.37%
4.75%
PI
annexin-V FITC

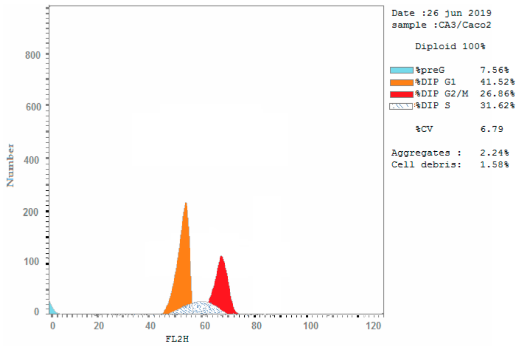
Date :26 jun 2019
sample :CA3/Caco2
Diploid 100%
%preG 7.56%
%DIP G1 41.52%
%DIP G2/M 26.86%
%DIP S 31.62%
%CV 6.79
Aggregates : 2.24%
Cell debris: 1.58%
Number
FL2H

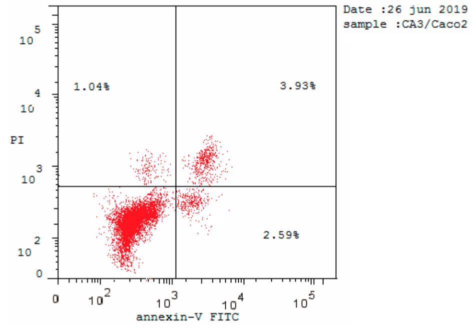
Date :26 jun 2019
sample :CA3/Caco2
1.04%
3.93%
2.59%
PI
annexin-V FITC

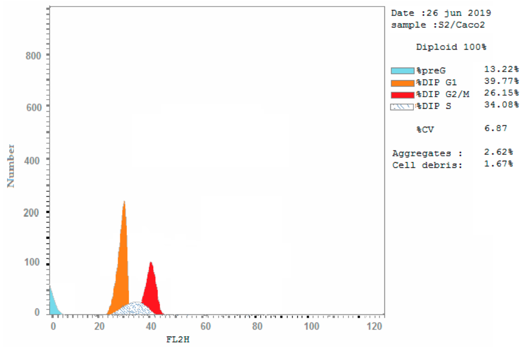
Date :26 jun 2019
sample :S2/Caco2
Diploid 100%
%preG 13.22%
%DIP G1 39.77%
%DIP G2/M 26.15%
%DIP S 34.08%
%CV 6.87
Aggregates : 2.62%
Cell debris: 1.67%
Number
FL2H

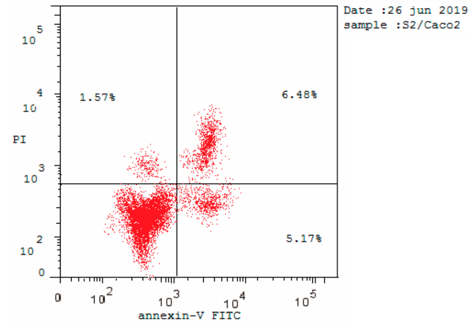
Date :26 jun 2019
sample :S2/Caco2
1.57%
6.48%
5.17%
PI
annexin-V FITC

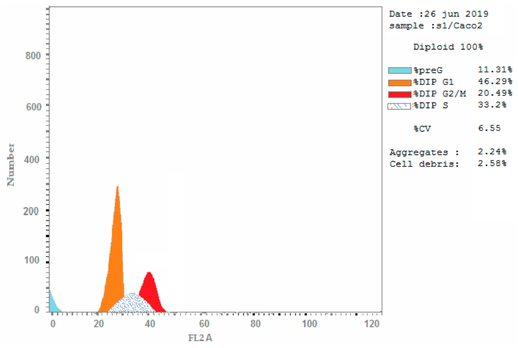
Date :26 jun 2019
sample :s1/Caco2
Diploid 100%
%preG 11.31%
%DIP G1 46.29%
%DIP G2/M 20.49%
%DIP S 33.2%
%CV 6.55
Aggregates : 2.24%
Cell debris: 2.58%
Number
FL2A

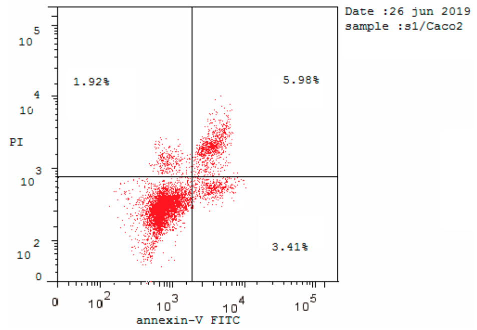
Date :26 jun 2019
sample :s1/Caco2
1.92%
5.98%
3.41%
PI
annexin-V FITC
