# Supplementary material for: Development of Semisynthetic Apoptosis-Inducing Agents Based on Natural Phenolic Acids Scaffold: Design, Synthesis and In-Vitro Biological Evaluation
Source: Molecules. 2022 Oct 9;27(19):6724. doi: 10.3390/molecules27196724 (PMC9571594; doi:10.3390/molecules27196724)
Supplement: Supplementary file 1 [file molecules-27-06724-s001.zip › Report RT-PCR (MMP-2 & 9).pdf]

Researcher : Dr/Noura email: *mob.*
Assay : MMP2,9 RT-PCR
Samples : 13 Samples
Cell lines : Caco2
Ref. : ----
Date : 19-10-2019
Reader : *Rotorgene RT- PCR system*
Kit used : Qiagen RNA extraction/**BioRad syber green PCR MMX**
Solvent : DMSO
Assay samples :Cell culture Lysate

Key for sample codes

Cpd. No.	Cpd. No.	Cpd. No.	Cpd. No.
**CA1**	**3**	FA1	**12**
**CA2**	**4**	FA2	**13**
**CA3**	**5**	FA3	**14**
**CA5**	**6**	FA5	**15**
**CA6**	**7**	FA7	**16**
**CA7**	**8**	FA8	**17**
**CA9**	**9**	FA9	**18**
**CA11**	**10**	FA11	**19**
**CA12**	**11**	FA12	**20**

RT-PCR
Report

Sample data				Results<br>Fold Change	
**Ser**	**code**	**cells**	conc	MMP2<br>FLD	MMP9<br>FLD
1	CA3	Caco2		0.59±0.016	0.44±0.009
2	CA6			0.16±0.004	0.29±0.006
3	CA9			0.83±0.022	0.70±0.014
4	CA11			0.33±0.008	0.55±0.011
5	CA12			0.54±0.014	0.30±0.006
6	FA3			0.84±0.022	0.73±0.015
7	FA5			0.59±0.016	0.37±0.007
8	FA8			0.15±0.004	0.17±0.003
9	FA9			0.42±0.011	0.52±0.010
10	FA12			0.68±0.018	0.45±0.009
11	1			0.16±0.004	0.24±0.004
12	2			0.76±0.020	0.62±0.012
13	cont.Caco2			1±0.027	1±0.020

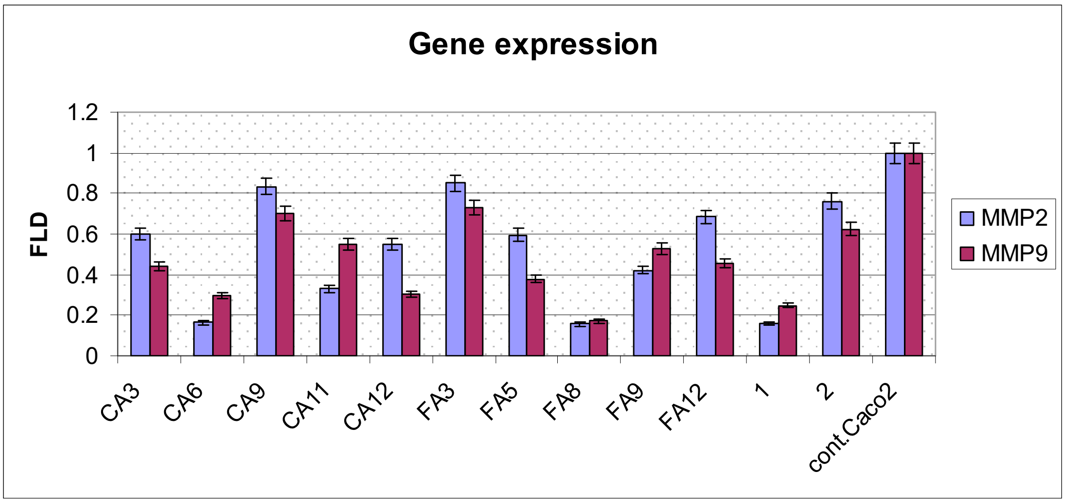

corbett RESEARCH
A Division of Corbett Life Science

14 Hilly Street Mortlake NSW 2137 Australia
T +61 2 9736 1320
F +61 2 9736 1364
W www.corbettlifescience.com

Quantitation Report

Experiment Information

Run Name	Run 2019-10-25 (2)
Run Start	2019-10-25 05:08:39 PM
Run Finish	2019-10-25 07:46:44 PM
Operator	EE
Notes	MMP9
Run On Software Version	Rotor-Gene 1.7.87
Run Signature	The Run Signature is valid.
Gain Green	10.
Gain Yellow	9.33

Quantitation data

Sample data			MMP2							
			Control cells			Test cells				FLD
Ser	Sample code	Conc uM	B Actin	MMP2	ΔCTC	B Actin	MMP2	ΔCTE	ΔΔ CT	2^ ΔΔCT
			HC	TC	TC-HC	HE	TE	TE-HE	ΔCTE-ΔCTC	Eamp=1.826
1	CA3		25.57	27.92	2.35	26.36	29.56	3.2	0.85	0.59941
2	CA6		25.57	27.92	2.35	25.81	31.17	5.36	3.01	0.163261
3	CA9		25.57	27.92	2.35	26.17	28.82	2.65	0.3	0.834737
4	CA11		25.57	27.92	2.35	26.22	30.41	4.19	1.84	0.330247
5	CA12		25.57	27.92	2.35	26.09	29.44	3.35	1	0.547645
6	FA3		25.57	27.92	2.35	25.94	28.56	2.62	0.27	0.849953
7	FA5		25.57	27.92	2.35	26.21	29.42	3.21	0.86	0.595812
8	FA8		25.57	27.92	2.35	25.94	31.38	5.44	3.09	0.155583
9	FA9		25.57	27.92	2.35	26.06	29.84	3.78	1.43	0.422721
10	FA12		25.57	27.92	2.35	25.78	28.76	2.98	0.63	0.684313
11	1		25.57	27.92	2.35	26.13	31.52	5.39	3.04	0.160338
12	2		25.57	27.92	2.35	25.91	28.71	2.8	0.45	0.762649
13	cont.Caco2		25.57	27.92	2.35	25.57	27.92	2.35	0	1

Sample data			MMP9							
			Control cells			Test cells				FLD
**Ser**	**Sample code**	**Conc uM**	B Actin	MMP9	ΔCTC	B Actin	MMP9	ΔCTE	ΔΔ CT	2^ ΔΔCT
			HC	TC	TC-HC	HE	TE	TE-HE	ΔCTE-ΔCTC	Eamp=1.826
1	CA3		25.57	27.92	2.35	26.36	30.07	3.71	1.36	0.440919
2	CA6		25.57	27.92	2.35	25.81	30.17	4.36	2.01	0.298115
3	CA9		25.57	27.92	2.35	26.17	29.11	2.94	0.59	0.700994
4	CA11		25.57	27.92	2.35	26.22	29.56	3.34	0.99	0.550953
5	CA12		25.57	27.92	2.35	26.09	30.41	4.32	1.97	0.305382
6	FA3		25.57	27.92	2.35	25.94	28.81	2.87	0.52	0.731172
7	FA5		25.57	27.92	2.35	26.21	30.17	3.96	1.61	0.379301
8	FA8		25.57	27.92	2.35	25.94	31.23	5.29	2.94	0.170289
9	FA9		25.57	27.92	2.35	26.06	29.47	3.41	1.06	0.528213
10	FA12		25.57	27.92	2.35	25.78	29.43	3.65	1.3	0.45714
11	1		25.57	27.92	2.35	26.13	30.79	4.66	2.31	0.248847
12	2		25.57	27.92	2.35	25.91	29.04	3.13	0.78	0.625215
13	cont.Caco2		25.57	27.92	2.35	25.57	27.92	2.35	0	1

This report generated by Rotor-Gene 6000 Series Software 1.7 (Build 87)
Copyright ©2000-2006 Corbett Research, a Division of Corbett Life Science. All rights reserved.
ISO 9001:2000 (Reg. No. QEC21313)

Quality System™
Quality Endorsed Company
ISO 9001 Lic 21313
SAI Global

[redacted]

primers

[redacted]

MMP2 F 5'- CAAAAACAAGAAGACATACATCTT-3'
MMP2 F 5'- CAAAAACAAGAAGACATACATCTT-3'

[redacted]

MMP9 F 5'- TGGGGGGCAACTCGGC-3'
MMP9 R 5'- GGAATGATCTAAGCCCAG-3'

[redacted]

β-actin F 5'-GTGACATCCACACCCAGAGG-3'
β-actin R 5'-ACAGGATGTCAAAACTGCCC-3'

[redacted]
