# Supplementary material for: Development of Semisynthetic Apoptosis-Inducing Agents Based on Natural Phenolic Acids Scaffold: Design, Synthesis and In-Vitro Biological Evaluation
Source: Molecules. 2022 Oct 9;27(19):6724. doi: 10.3390/molecules27196724 (PMC9571594; doi:10.3390/molecules27196724)
Supplement: Supplementary file 1 [file molecules-27-06724-s001.zip › Report _ RT-PCR.pdf]

Researcher : DR Moh.Abdelbasir email: m_a_baser@yahoo.com *mob.01*
Assay : RT-PCR
Samples : 12 Samples
Cell lines : ----
Ref. : ----
Date : 25-06-2019
Reader : *Rotorgene RT- PCR system*
Kit used : Qiagen RNA extraction/**BioRad syber green PCR MMX**
Solvent : DMSO
Assay samples :Cell culture Lysate

Key for sample codes

Cpd. No.	Cpd. No.	Cpd. No.	Cpd. No.
**CA1**	**3**	FA1	**12**
**CA2**	**4**	FA2	**13**
**CA3**	**5**	FA3	**14**
**CA5**	**6**	FA5	**15**
**CA6**	**7**	FA7	**16**
**CA7**	**8**	FA8	**17**
**CA9**	**9**	FA9	**18**
**CA11**	**10**	FA11	**19**
**CA12**	**11**	FA12	**20**

RT-PCR

Report

Sample data			Gene expression			
			Fold Change			
Ser	Sample code	conc	fld			
			Casp3	P53	Bcl2	Bax
1	s1/Caco2 (CA)		3.71±0.14	1.69±0.07	0.42±0.01	2.55±0.14
2	s2/Caco2 (FA)		7.82±0.28	3.98±0.15	0.63±0.04	5.24±0.26
3	CA3/Caco2		2.47±0.09	2.14±0.16	0.45±0.02	3.95±0.28
4	CA6/Caco2		12.62±0.81	9.29±0.64	0.69±0.04	6.76±0.26
5	CA9/Caco2		5.51±0.22	3.27±0.22	0.89±0.06	3.04±0.21
6	CA11/Caco2		16.39±0.84	14.52±0.88	0.47±0.02	15.57±0.14
7	CA12/Caco2		5.41±0.19	7.68±0.39	0.18±0.01	3.95±0.21
8	FA3/Caco2		9.78±0.34	11.18±0.72	0.40±0.02	5.91±0.36
9	FA5/Caco2		15.57±0.67	10.56±0.75	0.19±0.01	15.38±0.88
10	FA8/Caco2		5.04±0.26	3.14±0.18	0.77±0.05	5.84±0.24
11	FA9/Caco2		6.02±0.33	5.87±0.27	0.58±0.03	6.06±0.41
12	FA12/Caco2		4.64±0.18	3.98±0.16	0.37±0.02	3.33±0.16
***	cont.Caco2		1	1	1	1

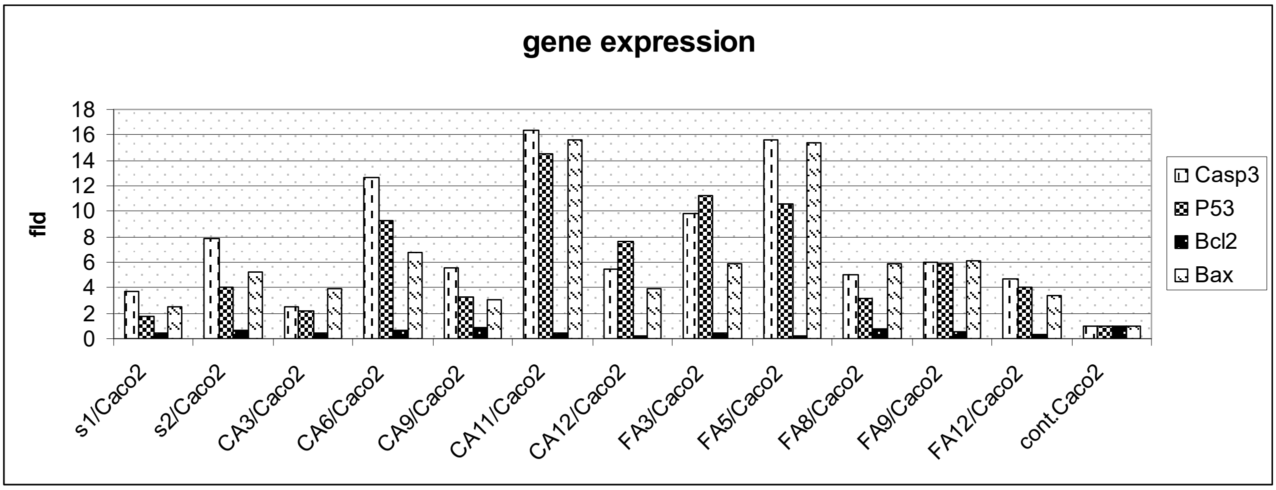

corbett RESEARCH
A Division of Corbett Life Science

14 Hilly Street Mortlake NSW 2137 Australia
T +61 2 9736 1320
F +61 2 9736 1364
W www.corbettlifescience.com

Quantitation Report

Experiment Information

Run Name	Run 2019-06-29 (1)
Run Start	2019-06-29 04:08:13 PM
Run Finish	2019-06-29 06:41:18 PM
Operator	ER
Notes	---
Run On Software Version	Rotor-Gene 1.7.87
Run Signature	The Run Signature is valid.
Gain Green	10.
Gain Yellow	9.33

Quantitation data

This report generated by Rotor-Gene 6000 Series Software 1.7 (Build 87)
Copyright ©2000-2006 Corbett Research, a Division of Corbett Life Science. All rights reserved.
ISO 9001:2000 (Reg. No. QEC21313)

[redacted]

primers

[redacted]

p53 F 5'-CCCCTCCTGGCCCCTGTCATCTTC-3',
p53 R 5'-GCAGCGCCTCACAACCTCCGTCAT-3'.

[redacted]

bcl-2 F 5′-CCTGTG GAT GAC TGA GTA CC-3′
bcl-2 R 5′-GAGACA GCC AGG AGA AAT CA-3′

[redacted]

CASP3-F 5′-TTC ATT ATT CAG GCC TGC CGA GG-3′
CASP3-R 5′-TTC TGA CAG GCC ATG TCA TCC TCA-3′

[redacted]

Bax F 5′-GTTTCA TCC AGG ATC GAG CAG-3′
Bax R 5′-CATCTT CTT CCA GAT GGT GA-3′

[redacted]

β-actin F 5′-GTGACATCCACACCCAGAGG-3′
β-actin R 5′-ACAGGATGTCAAAACTGCCC-3′

[redacted]

Quality System
Quality Endorsed Company
ISO 9001 Lic 21313
SAI Global

[redacted]

Sample data		Casp3							
		Control cells			Test cells				FLD
Ser	Sample code	B Actin	Casp3	ΔCTC	B Actin	Casp3	ΔCTE	ΔΔ CT	2^ ΔΔCT
		HC	TC	TC-HC	HE	TE	TE-HE	ΔCTE-ΔCTC	Eamp=1.891
1	s1/Caco2	26.42	34.21	7.79	25.75	31.48	5.73	-2.06	3.71522
2	s2/Caco2	26.42	34.21	7.79	26.16	30.72	4.56	-3.23	7.829132
3	CA3/Caco2	26.42	34.21	7.79	25.92	32.29	6.37	-1.42	2.471166
4	CA6/Caco2	26.42	34.21	7.79	26.04	29.85	3.81	-3.98	12.62503
5	CA9/Caco2	26.42	34.21	7.79	25.88	30.99	5.11	-2.68	5.514845
6	CA11/Caco2	26.42	34.21	7.79	26.31	29.71	3.4	-4.39	16.39366
7	CA12/Caco2	26.42	34.21	7.79	25.95	31.09	5.14	-2.65	5.41044
8	FA3/Caco2	26.42	34.21	7.79	26.13	30.34	4.21	-3.58	9.784884
9	FA5/Caco2	26.42	34.21	7.79	25.78	29.26	3.48	-4.31	15.57903
10	FA8/Caco2	26.42	34.21	7.79	26.02	31.27	5.25	-2.54	5.044249
11	FA9/Caco2	26.42	34.21	7.79	25.62	30.59	4.97	-2.82	6.029345
12	FA12/Caco2	26.42	34.21	7.79	26.16	31.54	5.38	-2.41	4.643298
13	cont.Caco2	26.42	34.21	7.79	26.42	34.21	7.79	0	1

[redacted]

Sample data		p53							
		Control cells			Test cells				FLD
Ser	Sample code	B Actin	p53	ΔCTC	B Actin	p53	ΔCTE	ΔΔ CT	2^ ΔΔCT
		HC	TC	TC-HC	HE	TE	TE-HE	ΔCTE-ΔCTC	Eamp=1.891
1	s1/Caco2	26.42	33.92	7.5	25.75	32.42	6.67	-0.83	1.696891
2	s2/Caco2	26.42	33.92	7.5	26.16	31.49	5.33	-2.17	3.984929
3	CA3/Caco2	26.42	33.92	7.5	25.92	32.22	6.3	-1.2	2.147978
4	CA6/Caco2	26.42	33.92	7.5	26.04	30.04	4	-3.5	9.29866
5	CA9/Caco2	26.42	33.92	7.5	25.88	31.52	5.64	-1.86	3.270742
6	CA11/Caco2	26.42	33.92	7.5	26.31	29.61	3.3	-4.2	14.52461
7	CA12/Caco2	26.42	33.92	7.5	25.95	30.25	4.3	-3.2	7.680914
8	FA3/Caco2	26.42	33.92	7.5	26.13	29.84	3.71	-3.79	11.18564
9	FA5/Caco2	26.42	33.92	7.5	25.78	29.58	3.8	-3.7	10.5623
10	FA8/Caco2	26.42	33.92	7.5	26.02	31.72	5.7	-1.8	3.148073
11	FA9/Caco2	26.42	33.92	7.5	25.62	30.34	4.72	-2.78	5.877633
12	FA12/Caco2	26.42	33.92	7.5	26.16	31.49	5.33	-2.17	3.984929
13	cont.Caco2	26.42	33.92	7.5	26.42	33.92	7.5	0	1

[redacted]

Sample data		Bcl2							
		Control cells			Test cells				FLD
Ser	Sample code	B Actin	Bcl2	ΔCTC	B Actin	Bcl2	ΔCTE	ΔΔ CT	2^ ΔΔCT
		HC	TC	TC-HC	HE	TE	TE-HE	ΔCTE-ΔCTC	Eamp=1.891
1	s1/Caco2	26.42	30.47	4.05	25.75	31.15	5.4	1.35	0.423123
2	s2/Caco2	26.42	30.47	4.05	26.16	30.92	4.76	0.71	0.636135
3	CA3/Caco2	26.42	30.47	4.05	25.92	31.19	5.27	1.22	0.45966
4	CA6/Caco2	26.42	30.47	4.05	26.04	30.66	4.62	0.57	0.695482
5	CA9/Caco2	26.42	30.47	4.05	25.88	30.11	4.23	0.18	0.891652
6	CA11/Caco2	26.42	30.47	4.05	26.31	31.54	5.23	1.18	0.471524
7	CA12/Caco2	26.42	30.47	4.05	25.95	32.62	6.67	2.62	0.188394
8	FA3/Caco2	26.42	30.47	4.05	26.13	31.61	5.48	1.43	0.402097
9	FA5/Caco2	26.42	30.47	4.05	25.78	32.42	6.64	2.59	0.19203
10	FA8/Caco2	26.42	30.47	4.05	26.02	30.46	4.44	0.39	0.779992
11	FA9/Caco2	26.42	30.47	4.05	25.62	30.51	4.89	0.84	0.58557
12	FA12/Caco2	26.42	30.47	4.05	26.16	31.76	5.6	1.55	0.372502
13	cont.Caco2	26.42	30.47	4.05	26.42	30.47	4.05	0	1

[redacted]

Sample data		Bax							
		Control cells			Test cells				FLD
Ser	Sample code	B Actin	Bax	ΔCTC	B Actin	Bax	ΔCTE	ΔΔ CT	2^ ΔΔCT
		HC	TC	TC-HC	HE	TE	TE-HE	ΔCTE-ΔCTC	Eamp=1.891
1	s1/Caco2	26.42	34.71	8.29	25.75	32.57	6.82	-1.47	2.551153
2	s2/Caco2	26.42	34.71	8.29	26.16	31.85	5.69	-2.6	5.240805
3	CA3/Caco2	26.42	34.71	8.29	25.92	32.05	6.13	-2.16	3.959622
4	CA6/Caco2	26.42	34.71	8.29	26.04	31.33	5.29	-3	6.761991
5	CA9/Caco2	26.42	34.71	8.29	25.88	32.42	6.54	-1.75	3.04937
6	CA11/Caco2	26.42	34.71	8.29	26.31	30.29	3.98	-4.31	15.57903
7	CA12/Caco2	26.42	34.71	8.29	25.95	32.08	6.13	-2.16	3.959622
8	FA3/Caco2	26.42	34.71	8.29	26.13	31.63	5.5	-2.79	5.915199
9	FA5/Caco2	26.42	34.71	8.29	25.78	29.78	4	-4.29	15.38178
10	FA8/Caco2	26.42	34.71	8.29	26.02	31.54	5.52	-2.77	5.840305
11	FA9/Caco2	26.42	34.71	8.29	25.62	31.08	5.46	-2.83	6.067881
12	FA12/Caco2	26.42	34.71	8.29	26.16	32.56	6.4	-1.89	3.333857
13	cont.Caco2	26.42	34.71	8.29	26.42	34.71	8.29	0	1
