# Supplementary material for: Development of Semisynthetic Apoptosis-Inducing Agents Based on Natural Phenolic Acids Scaffold: Design, Synthesis and In-Vitro Biological Evaluation
Source: Molecules. 2022 Oct 9;27(19):6724. doi: 10.3390/molecules27196724 (PMC9571594; doi:10.3390/molecules27196724)

Science Way
For Scientific Researches

Viability assay

Test code: T-4-019-5

Institute / Researcher: **prof.Dr. nora abo rehab**

Experiment : **functional assay (MTT)**

(viability/cytotoxicity)

samples number: **2**

experiment design: viability against HepG2, Panc 1, Caco2, A549, Mcf7 and vero cells

laboratory comments:

Viability assay

MTT protocol

Determination of sample cytotoxicity on cells (MTT protocol)

1-the 96 well tissue culture plate was inoculated with 1 X 10^5 cells / ml (100 ul / well) and incubated at 37°C for 24 hours to develop a complete monolayer sheet.

2- Growth medium was decanted from 96 well micro titer plates after confluent sheet of cells were formed, cell monolayer was washed twice with wash media.

3- two-fold dilutions of tested sample was made in RPMI medium with 2% serum (maintenance medium).

4- 0.1 ml of each dilution was tested in different wells leaving 3 wells as control, receiving only maintenance medium.

5- Plate was incubated at 37°C and examined. Cells were checked for any physical signs of toxicity, e.g. partial or complete loss of the monolayer, rounding, shrinkage, or cell granulation.

6- MTT solution was prepared (5mg/ml in PBS) (BIO BASIC CANADA INC).

8- 20ul MTT solution were added to each well. Place on a shaking table, 150rpm for 5 minutes, to thoroughly mix the MTT into the media.

9) Incubate (37C, 5% CO2) for 1-5 hours to allow the MTT to be metabolized.

10) Dump off the media. (dry plate on paper towels to remove residue if necessary.

11) Resuspend formazan (MTT metabolic product) in 200ul DMSO. Place on a shaking table, 150rpm for 5 minutes, to thoroughly mix the formazan into the solvent.

12) Read optical density at 560nm and subtract background at 620nm. Optical density should be directly correlated with cell quantity.

ID	Conc. ug/ml	O.D			Mean O.D	ST.E	Viability %	Toxicity %	IC50
A549	dilution	0.256	0.284	0.267	0.269	0.008145	100	0	ug
1	500	0.063	0.052	0.058	0.057667	0.00318	21.43742255	78.56257745	316.639
	250	0.13	0.119	0.131	0.126667	0.003844	47.08798017	52.91201983	
	125	0.262	0.251	0.263	0.258667	0.003844	96.15861214	3.841387856	
	62.5	0.264	0.27	0.265	0.266333	0.001856	99.0086741	0.991325898	
	31.25	0.272	0.269	0.265	0.268667	0.002028	99.87608426	0.123915737	
	15.625	0.263	0.274	0.264	0.267	0.003512	99.25650558	0.743494424	
2	500	0.054	0.066	0.074	0.064667	0.005812	24.03965304	75.96034696	314.441
	250	0.113	0.099	0.118	0.11	0.005686	40.89219331	59.10780669	
	125	0.254	0.253	0.267	0.258	0.004509	95.91078067	4.089219331	
	62.5	0.268	0.27	0.263	0.267	0.002082	99.25650558	0.743494424	
	31.25	0.275	0.264	0.259	0.266	0.004726	98.88475836	1.115241636	
	15.625	0.267	0.267	0.266	0.266667	0.000333	99.13258984	0.867410161	

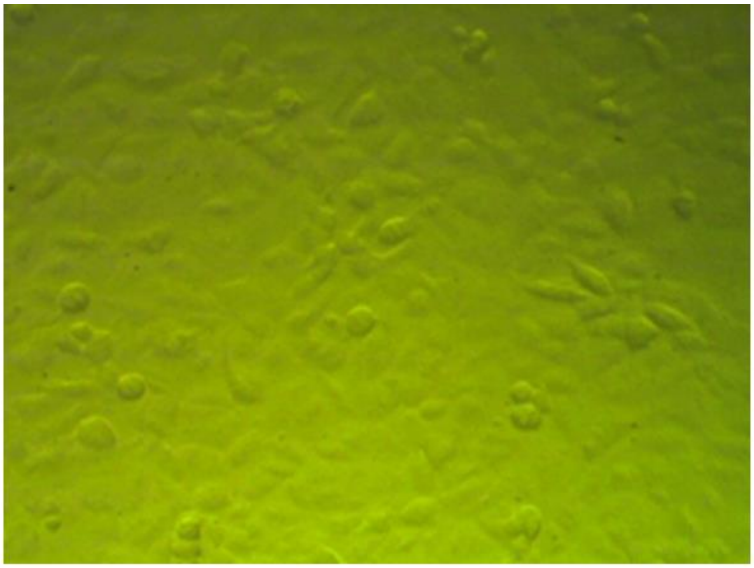

control
A549 cells

Organism : *Homo sapiens*, human
Tissue : lung
Cell Type : epithelial
Culture Properties : adherent
Disease : Carcinoma

Effect of comp. 1 on A549 cells at different concentration

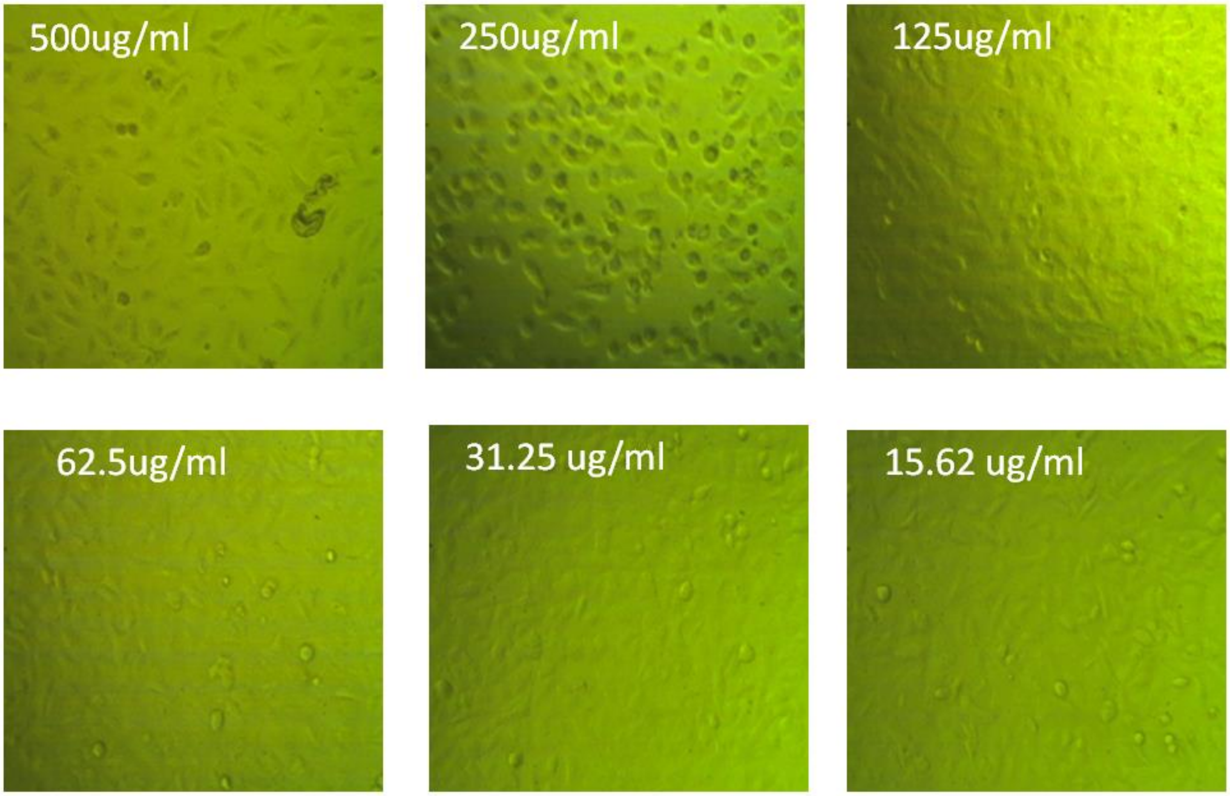

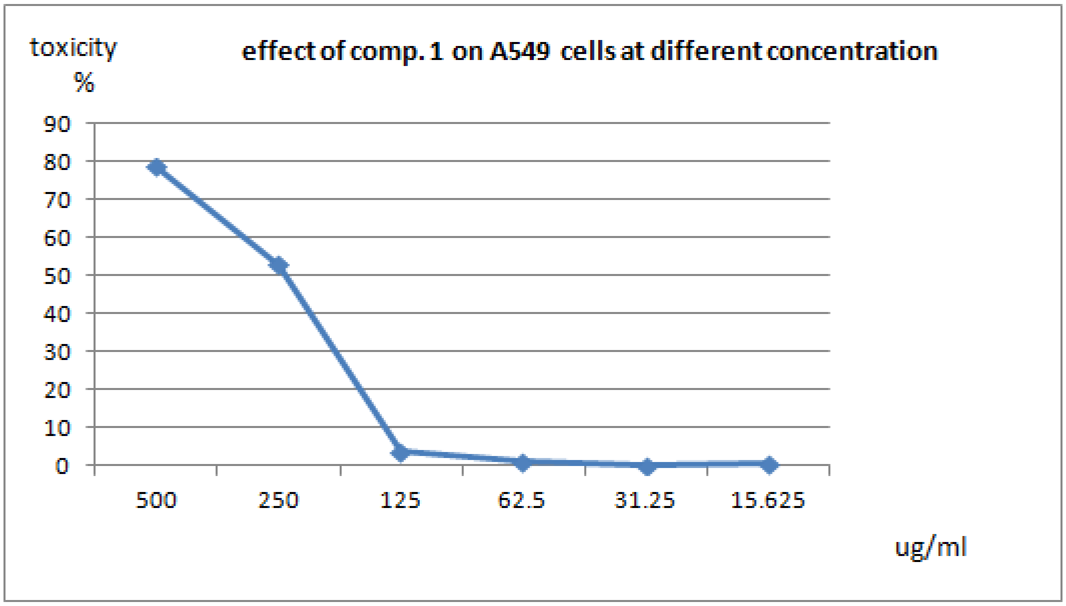

Effect of comp. 2 on A549 cells at different concentration

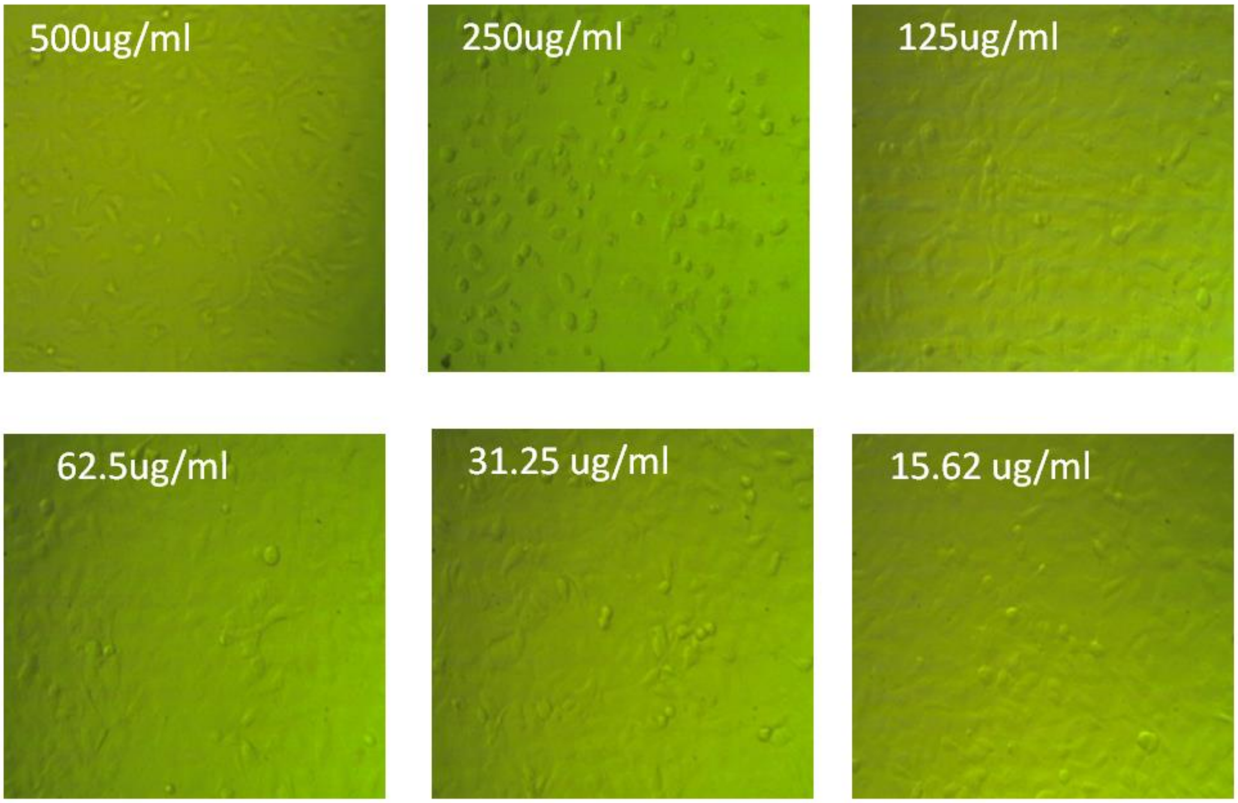

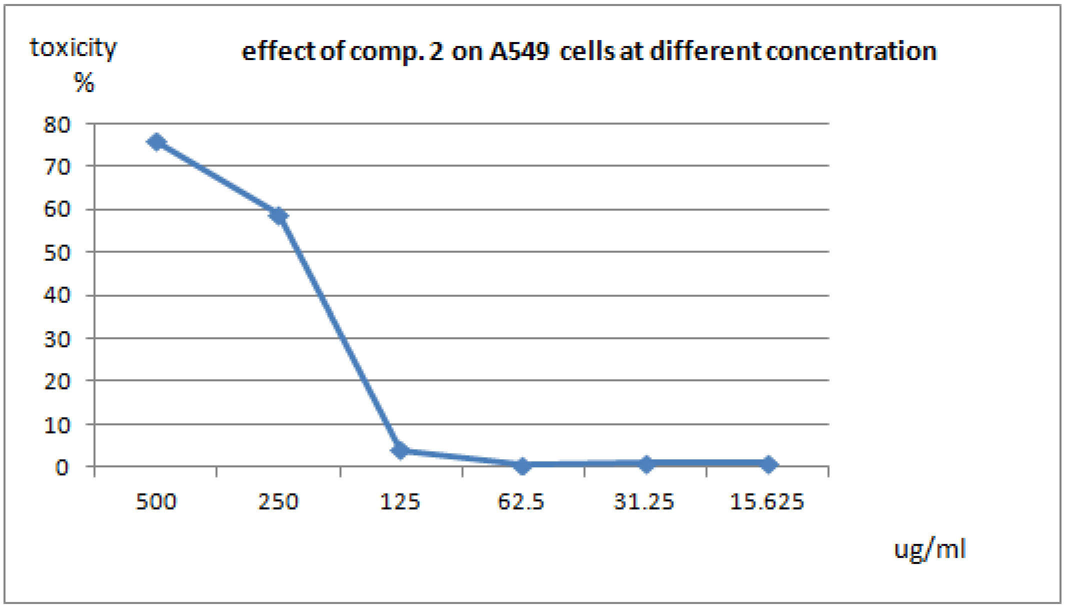

شركة ساينس واى للابحاث و الاستشارات العلميه ب.ض/810-510-523 س.ت/ 93150
المعمل الرئيسى / المقطم - الهضبه العليا - ش 9
للتدريب و عمل الابحاث / مدينة نصر - الحى العاشر - برج الزهور ب- امام مسجد الخزان

ID	Conc. ug/ml	O.D			Mean O.D	ST.E	Viability %	Toxicity %	IC50
Caco2	dilution	0.336	0.348	0.357	0.347	0.006083	100	0	ug
1	500	0.041	0.036	0.047	0.041333	0.00318	11.91162344	88.08837656	81.173
	250	0.074	0.066	0.069	0.069667	0.002333	20.07684918	79.92315082	
	125	0.089	0.092	0.087	0.089333	0.001453	25.74447646	74.25552354	
	62.5	0.154	0.169	0.168	0.163667	0.004842	47.16618636	52.83381364	
	31.25	0.302	0.314	0.316	0.310667	0.004372	89.52929875	10.47070125	
	15.625	0.347	0.35	0.343	0.346667	0.002028	99.90393852	0.096061479	
2	500	0.045	0.063	0.055	0.054333	0.005207	15.65802113	84.34197887	96.402
	250	0.042	0.037	0.044	0.041	0.002082	11.81556196	88.18443804	
	125	0.097	0.108	0.101	0.102	0.003215	29.39481268	70.60518732	
	62.5	0.274	0.263	0.269	0.268667	0.00318	77.42555235	22.57444765	
	31.25	0.314	0.312	0.317	0.314333	0.001453	90.58597502	9.414024976	
	15.625	0.341	0.351	0.346	0.346	0.002887	99.71181556	0.288184438	

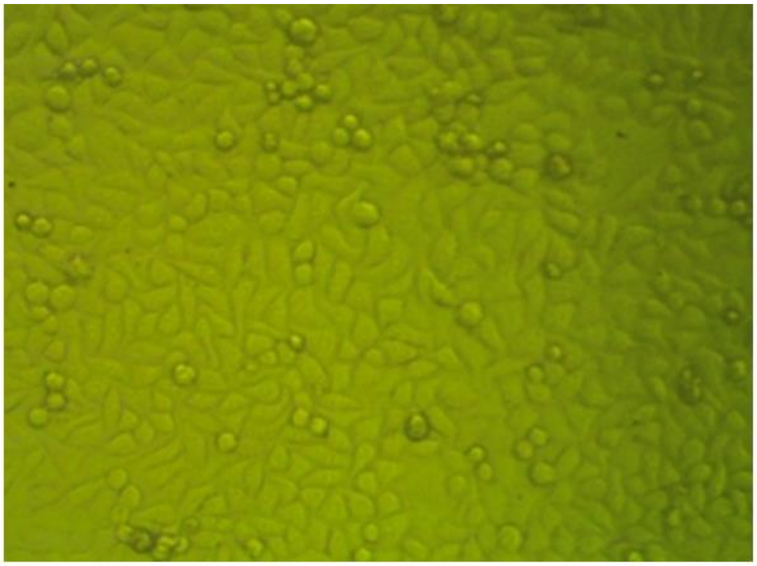

control
Caco2 cells

Organism : *Homo sapiens*, human
Tissue : Colon
Cell Type : epithelial
Culture Properties : adherent
Disease : Colorectal adenocarcinoma
ATCC : ATB-37

Effect of comp. 1 on Caco2 cells at different concentration

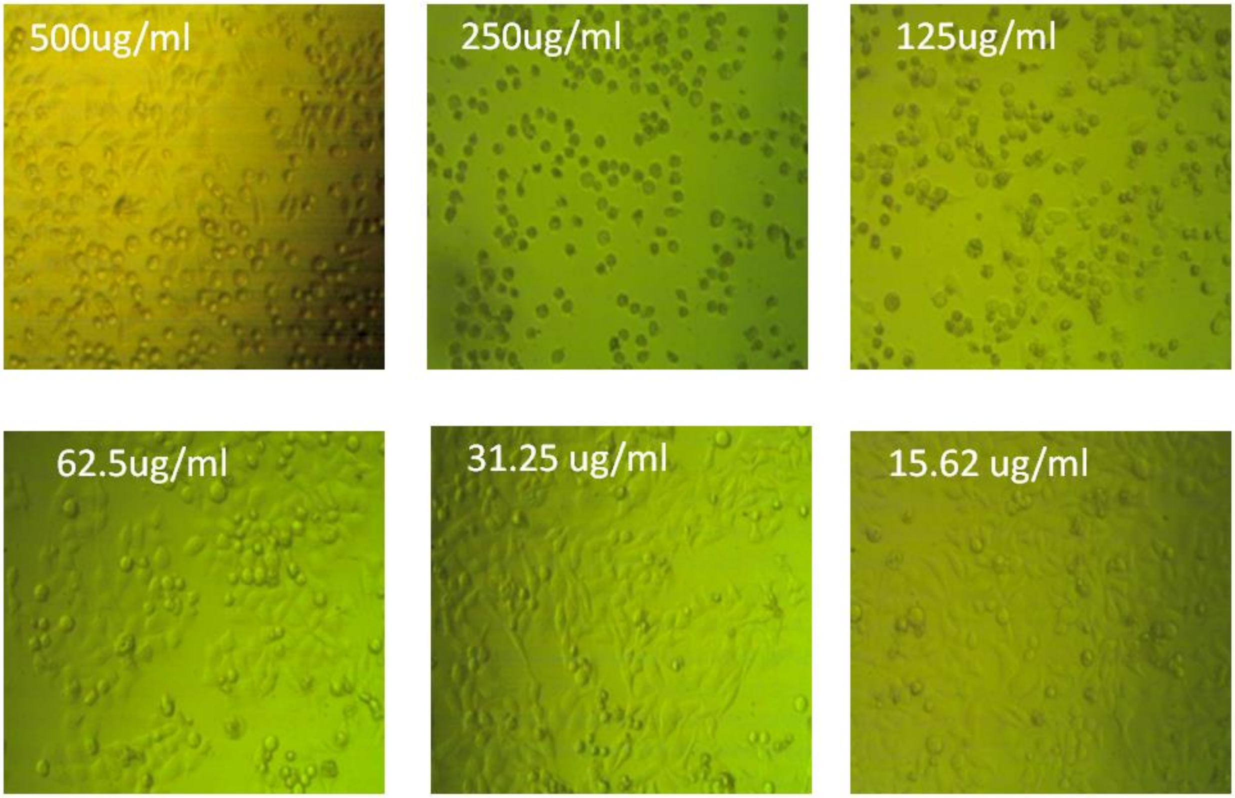

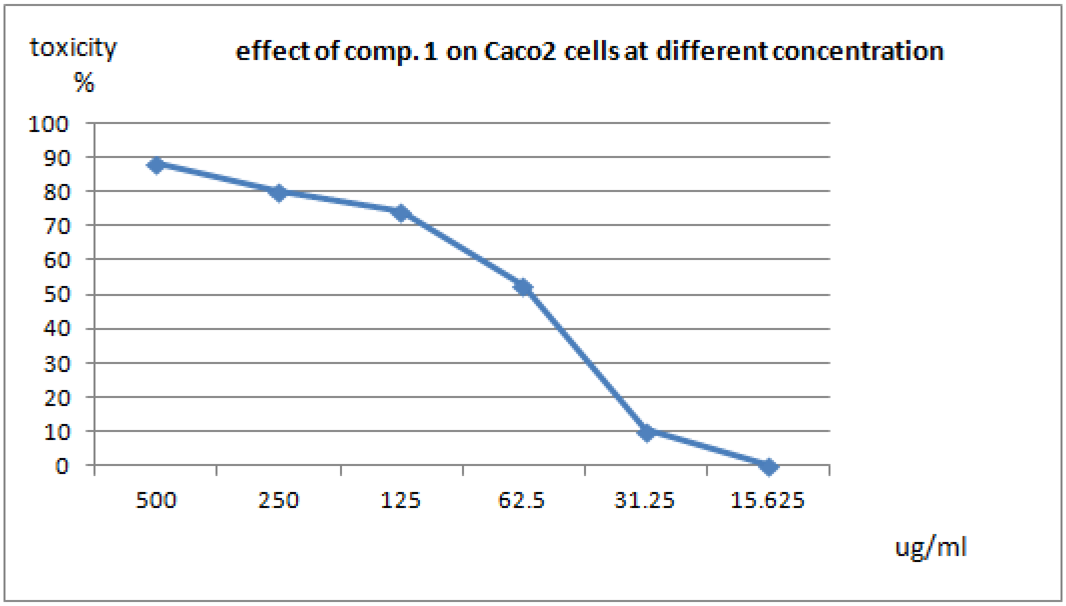

شركة ساينس واى للابحاث و الاستشارات العلميه ب.ض/810-510-523 س.ت/ 93150
المعمل الرئيسى / المقطم - الهضبه العليا - ش 9
للتدريب و عمل الابحاث / مدينة نصر - الحى العاشر - برج الزهور ب- امام مسجد الخزان

Effect of comp. 2 on Caco2 cells at different concentration

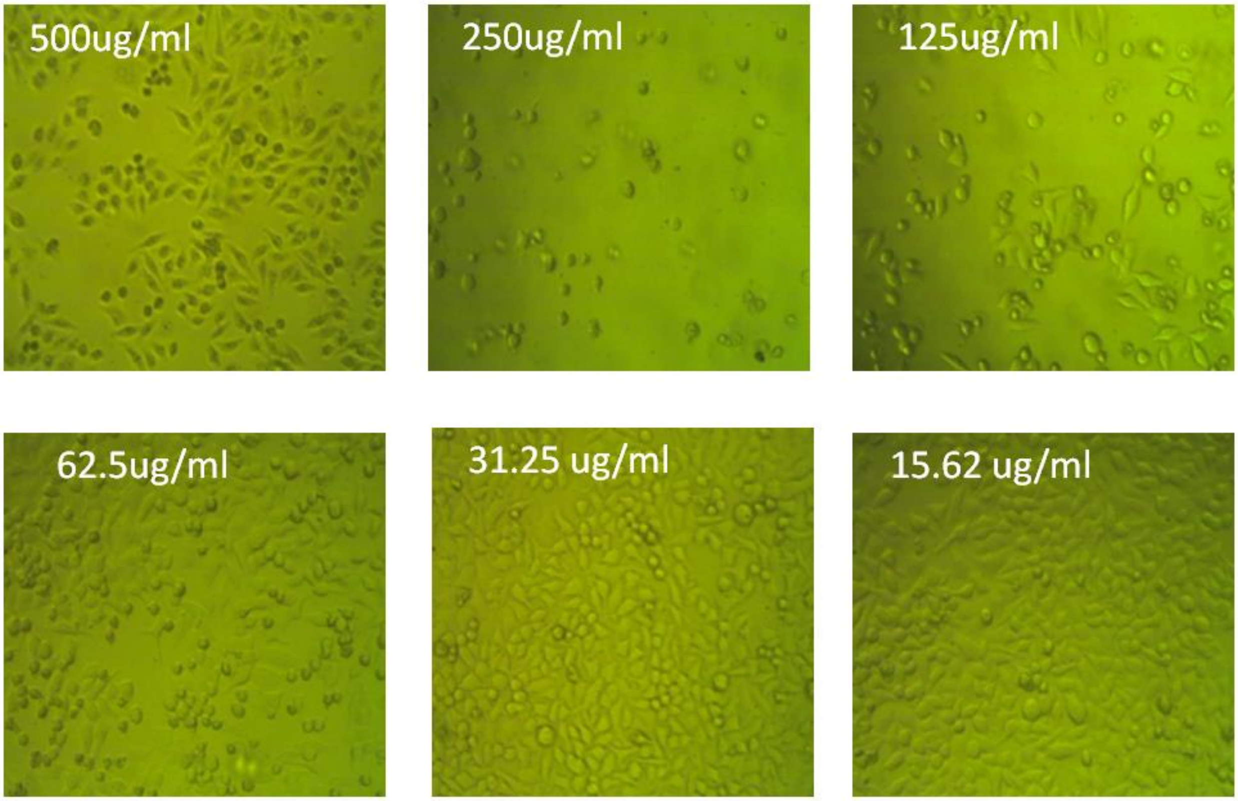

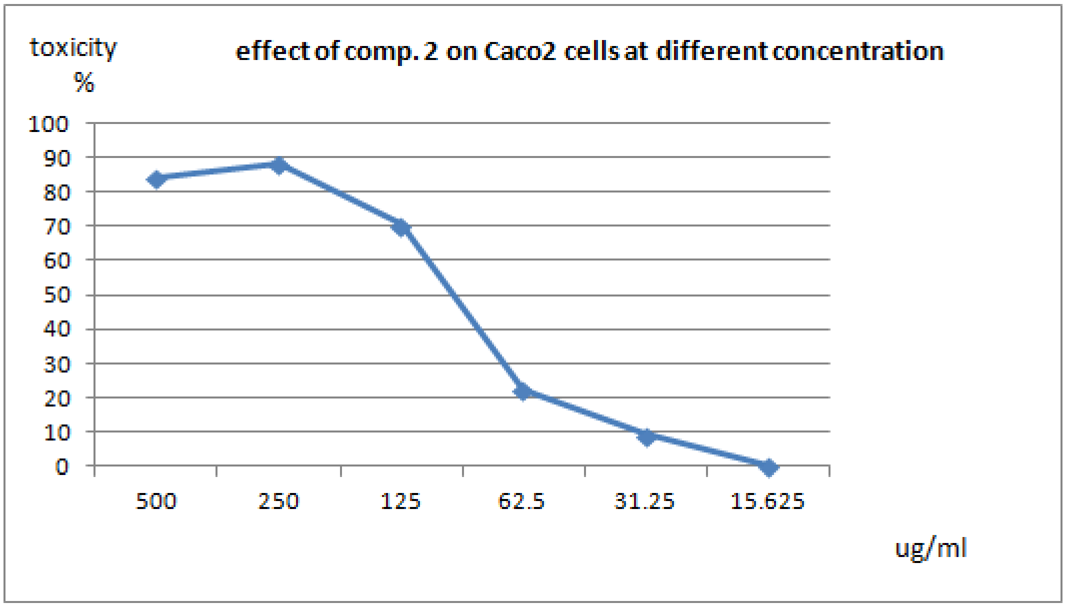

ID	Conc. ug/ml	O.D			Mean O.D	ST.E	Viability %	Toxicity %	IC50
HepG2	dilution	0.323	0.341	0.326	0.33	0.005568	100	0	ug
1	500	0.06	0.057	0.042	0.053	0.005568	16.06060606	83.93939394	201.181
	250	0.097	0.083	0.096	0.092	0.004509	27.87878788	72.12121212	
	125	0.284	0.277	0.294	0.285	0.004933	86.36363636	13.63636364	
	62.5	0.324	0.331	0.33	0.328333	0.002186	99.49494949	0.505050505	
	31.25	0.329	0.326	0.334	0.329667	0.002333	99.8989899	0.101010101	
	15.625	0.326	0.331	0.331	0.329333	0.001667	99.7979798	0.202020202	

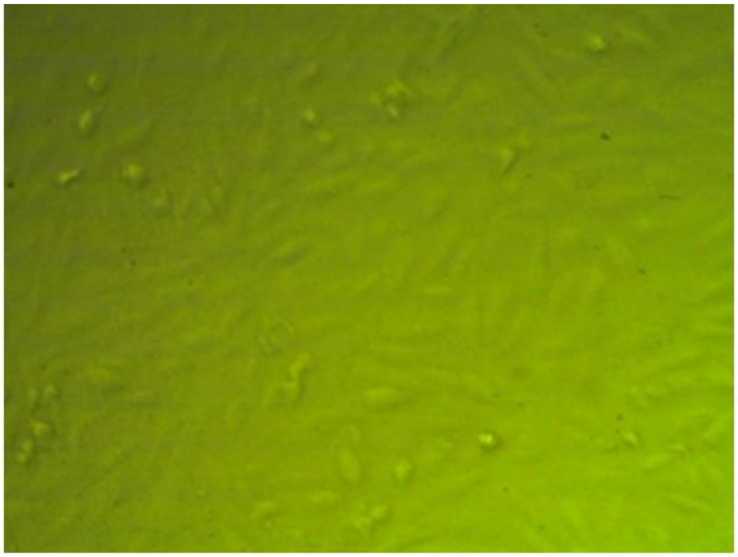

control
HepG2 cells

Organism : *Homo sapiens*, human
Tissue : liver
Cell Type : epithelial
Culture Properties : adherent
Disease : hepatocellular carcinoma

Effect of comp. 1 on HepG2 cells at different concentration

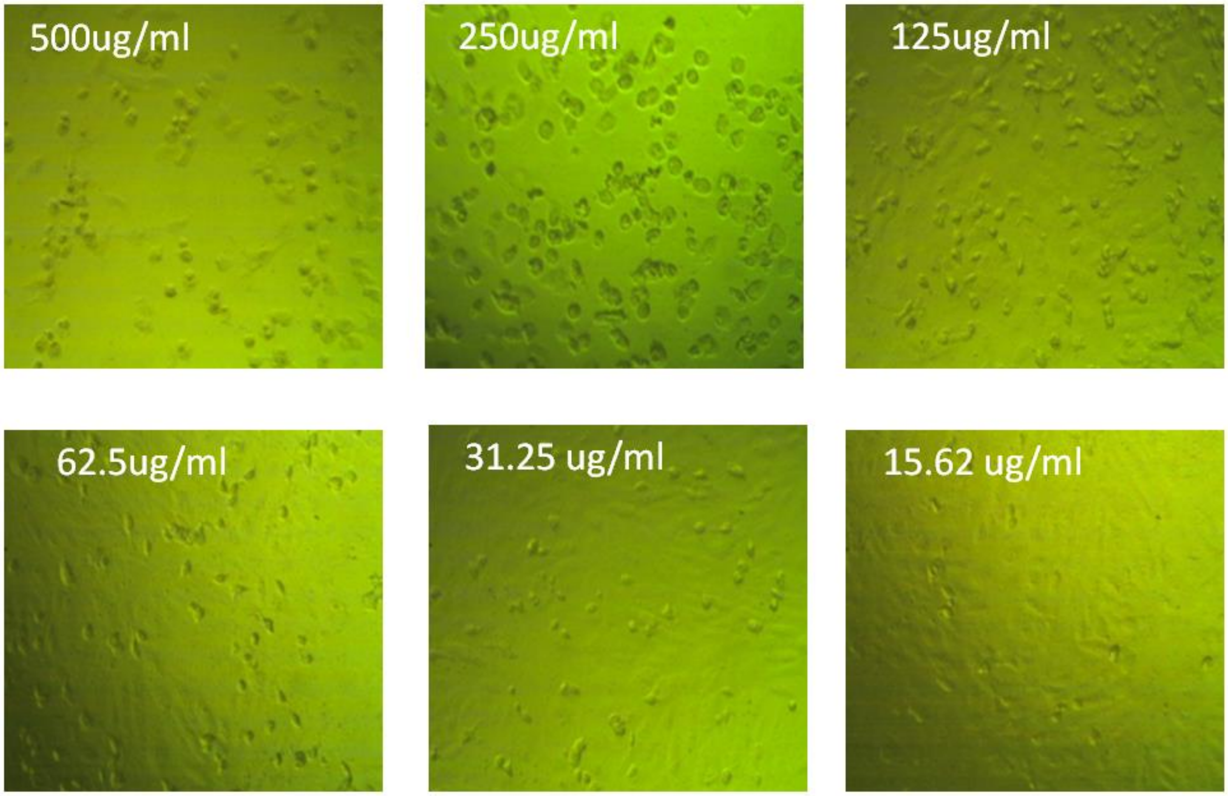

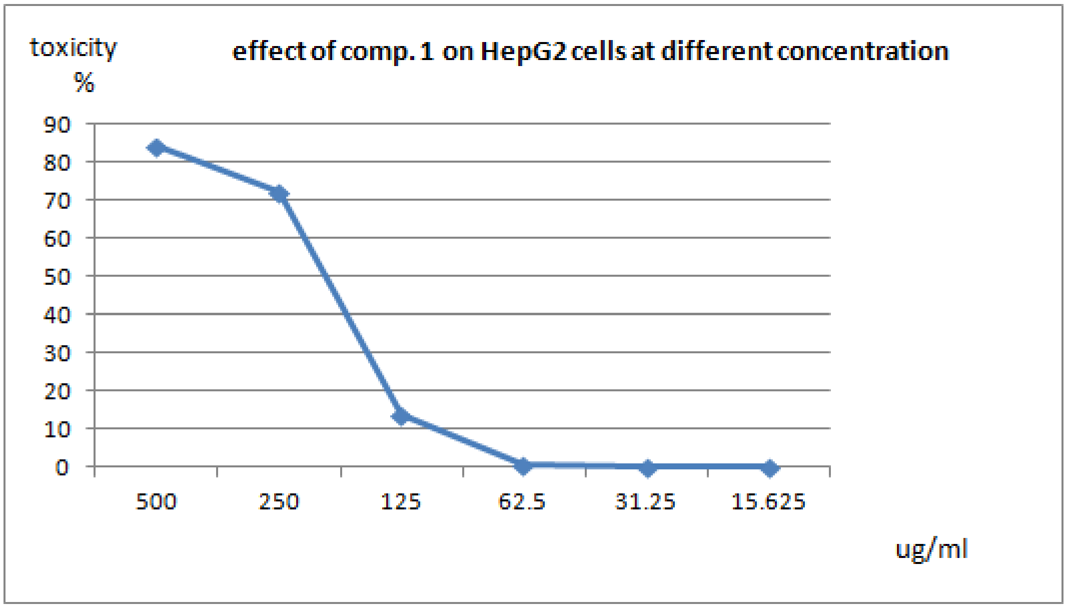

ID	Conc. ug/ml	O.D			Mean O.D	ST.E	Viability %	Toxicity %	IC50
Mcf7	dilution	0.299	0.316	0.318	0.311	0.006028	100	0	ug
2	500	0.036	0.04	0.033	0.036333	0.002028	11.68274384	88.31725616	202.921
	250	0.097	0.094	0.083	0.091333	0.004256	29.3676313	70.6323687	
	125	0.277	0.248	0.267	0.264	0.008505	84.88745981	15.11254019	
	62.5	0.31	0.305	0.311	0.308667	0.001856	99.24973205	0.750267953	
	31.25	0.306	0.314	0.307	0.309	0.002517	99.35691318	0.643086817	
	15.625	0.316	0.299	0.306	0.307	0.004933	98.71382637	1.286173633	

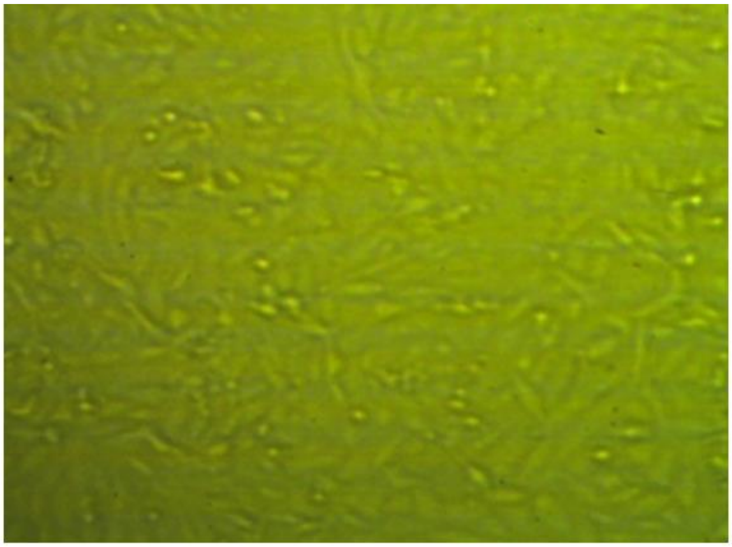

control
Mcf7 cells

Organism : *Homo sapiens*, human
Tissue : mammary gland, breast; derived from metastatic site: pleural effusion
Cell Type : epithelial
Culture Properties : adherent
Disease : adenocarcinoma
ATCC : HTB-22

شركة ساينس واى للابحاث و الاستشارات العلميه ب.ض/810-510-523 س.ت/ 93150
المعمل الرئيسى / المقطم - الهضبه العليا - ش 9
للتدريب و عمل الابحاث / مدينة نصر - الحى العاشر - برج الزهور ب- امام مسجد الخزان

Effect of comp. 2 on Mcf7 cells at different concentration

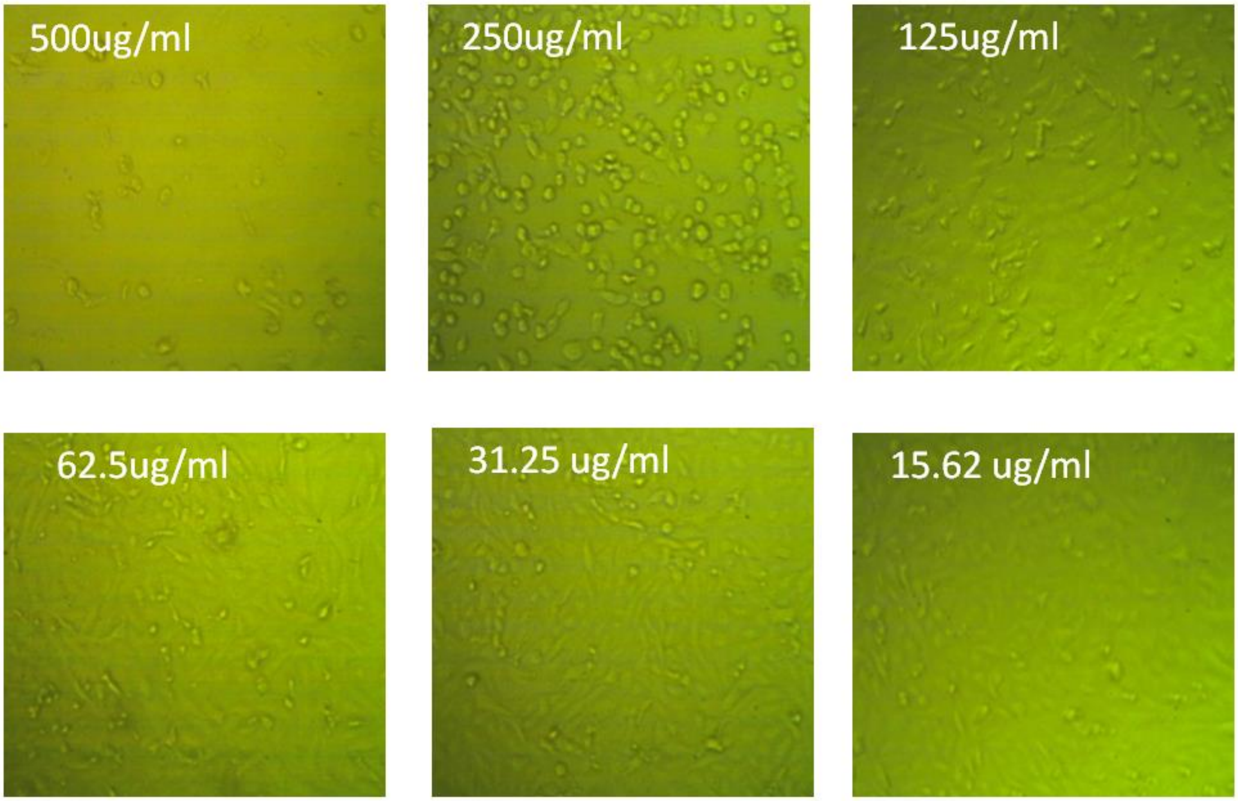

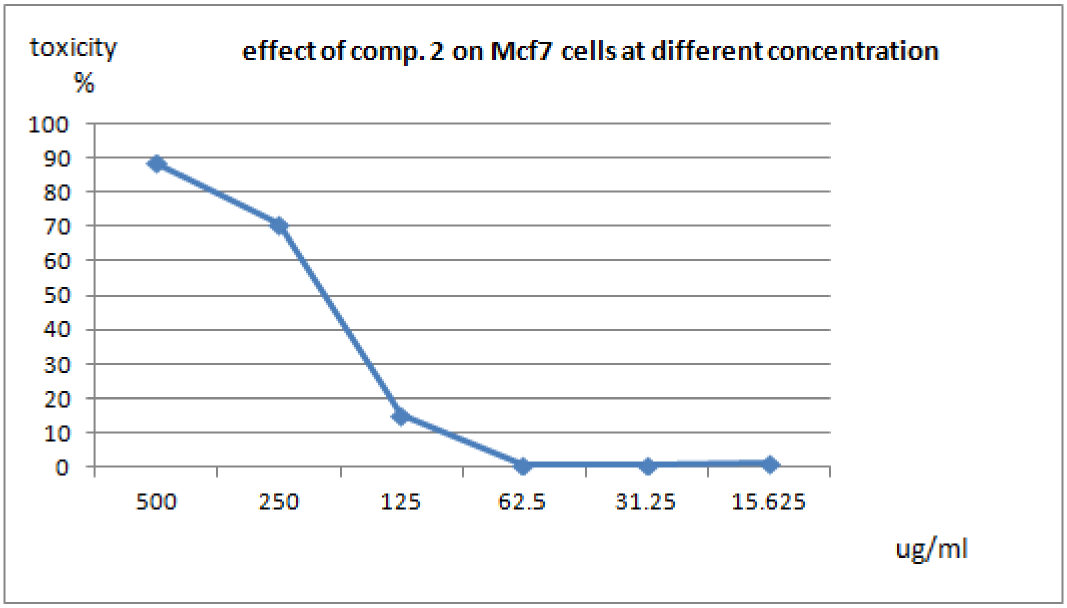

ID	Conc. ug/ml	O.D			Mean O.D	ST.E	Viability %	Toxicity %	IC50
Panc1	dilution	0.374	0.361	0.369	0.368	0.003786	100	0	ug
1	500	0.111	0.105	0.112	0.109333	0.002186	29.71014493	70.28985507	250.824
	250	0.197	0.152	0.184	0.177667	0.013371	48.27898551	51.72101449	
	125	0.336	0.341	0.336	0.337667	0.001667	91.75724638	8.242753623	
	62.5	0.364	0.373	0.365	0.367333	0.002848	99.81884058	0.18115942	
	31.25	0.363	0.375	0.372	0.37	0.003606	100.5434783	0	
	15.625	0.374	0.361	0.369	0.368	0.003786	100	0	
2	500	0.11	0.102	0.103	0.105	0.002517	28.5326087	71.4673913	195.396
	250	0.093	0.097	0.085	0.091667	0.003528	24.90942029	75.09057971	
	125	0.311	0.325	0.319	0.318333	0.004055	86.50362319	13.49637681	
	62.5	0.373	0.365	0.369	0.369	0.002309	100.2717391	0	
	31.25	0.364	0.369	0.368	0.367	0.001528	99.72826087	0.27173913	
	15.625	0.371	0.362	0.373	0.368667	0.003383	100.1811594	0	

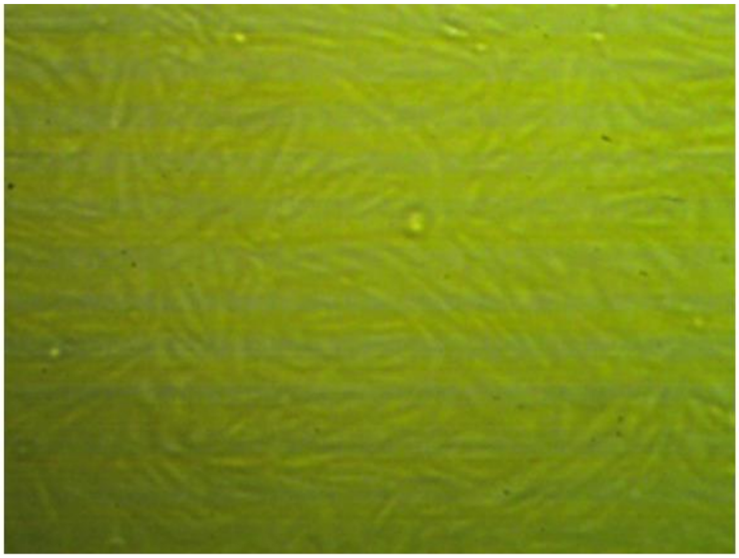

control
PANC1 cells

Organism : *Homo sapiens*, human
Tissue : pancreas/duct
Cell Type : epithelial
Culture Properties : adherent
Disease : epithelioid carcinoma

Effect of comp. 1 on Panc1 cells at different concentration

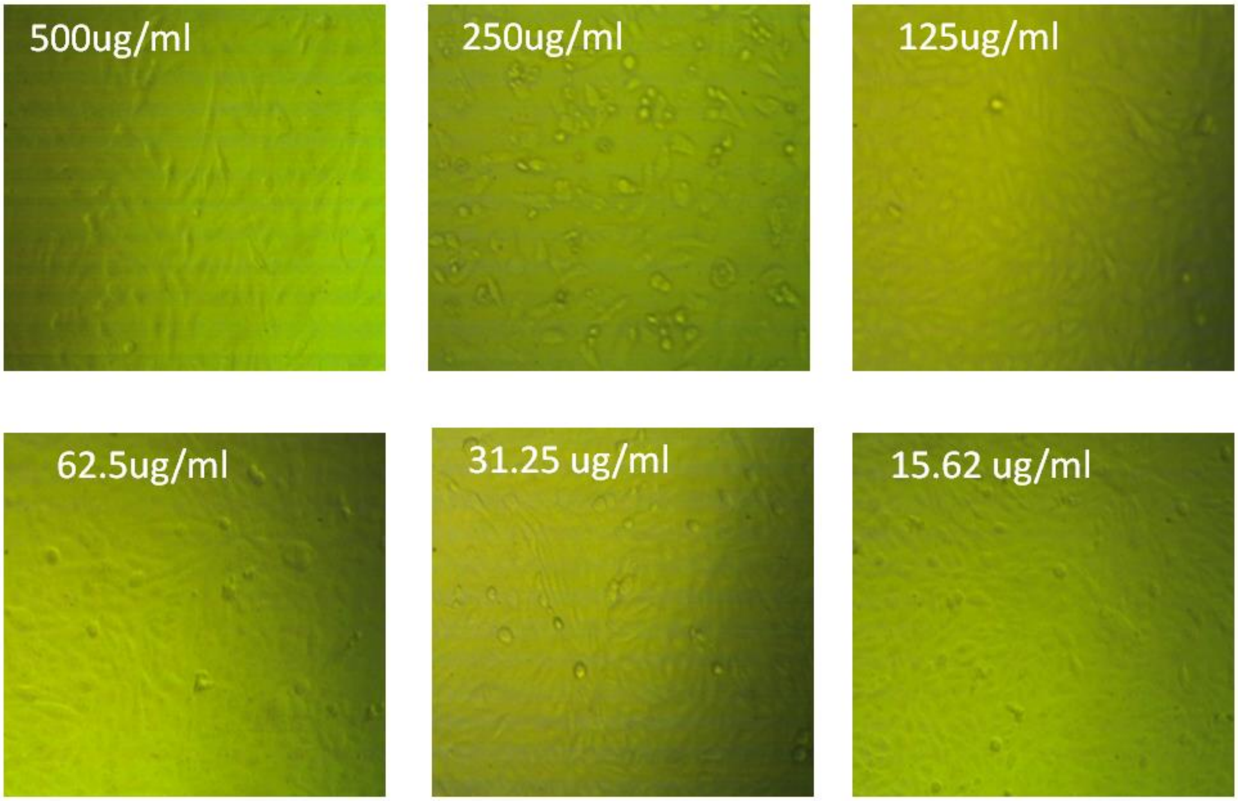

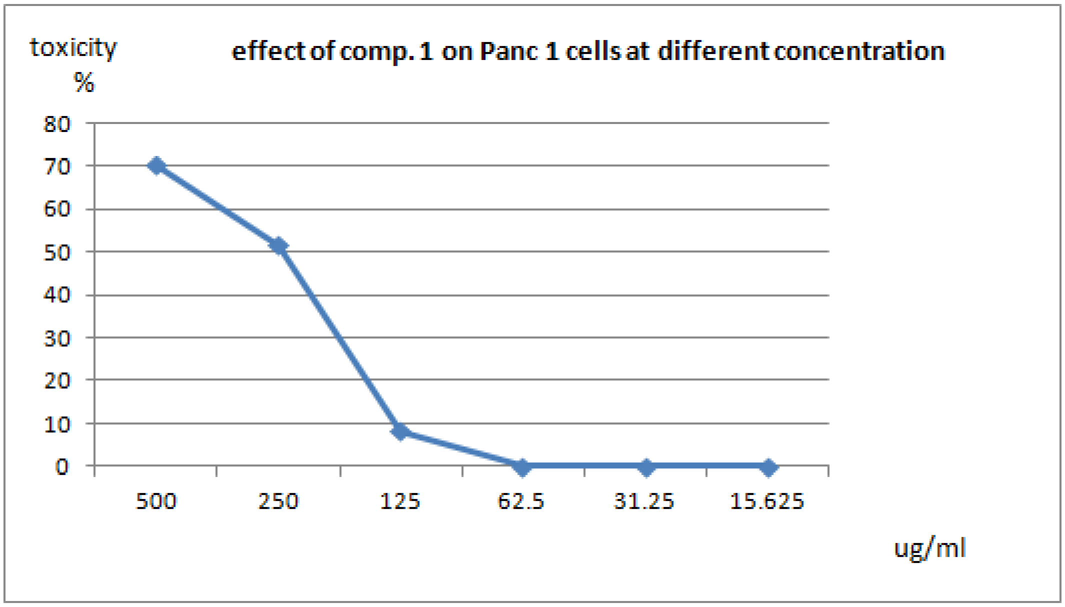

شركة ساينس واى للابحاث و الاستشارات العلميه ب.ض/810-510-523 س.ت/ 93150
المعمل الرئيسى / المقطم - الهضبه العليا - ش 9
للتدريب و عمل الابحاث / مدينة نصر - الحى العاشر - برج الزهور ب- امام مسجد الخزان

Effect of comp. 2 on Panc1 cells at different concentration

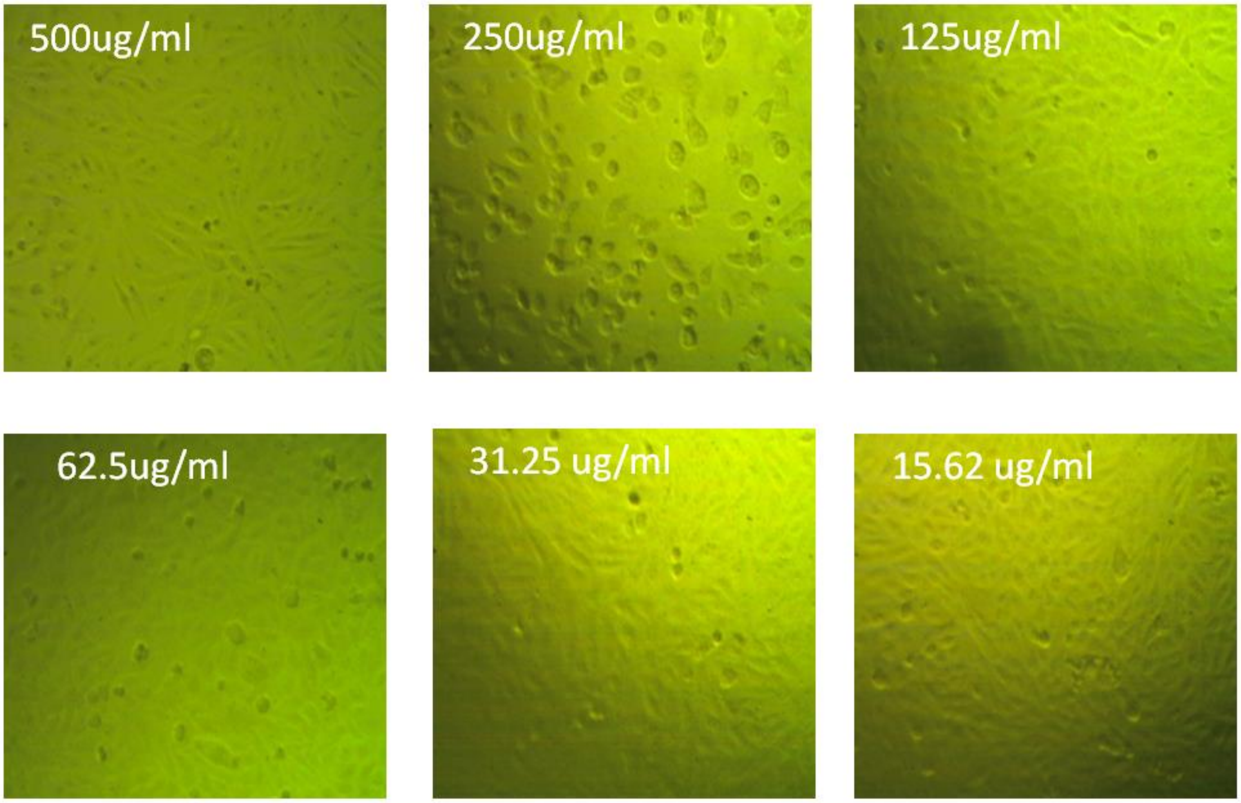

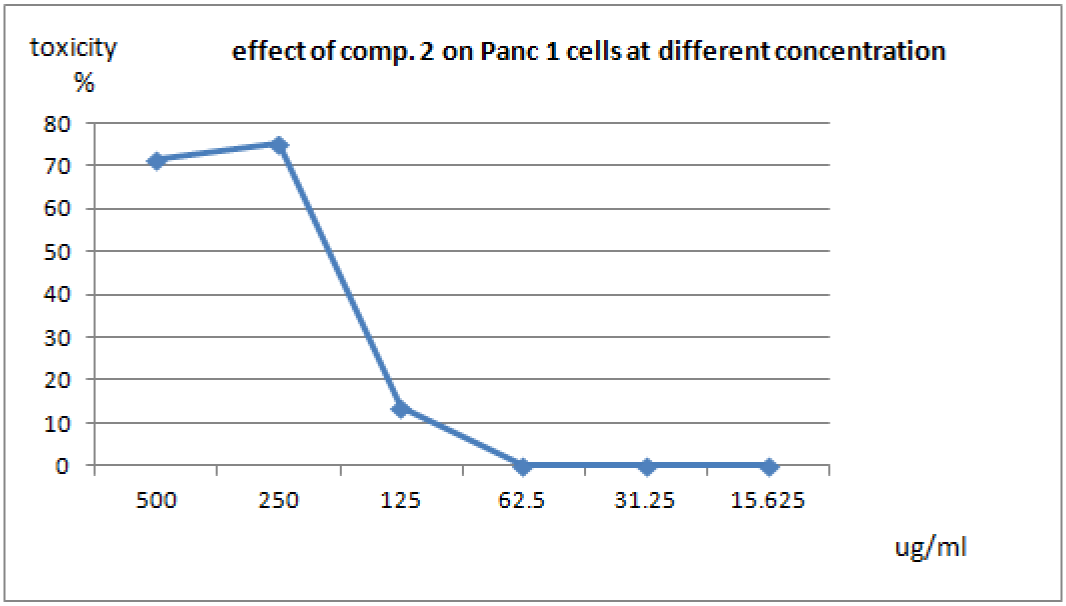

شركة ساينس واى للابحاث و الاستشارات العلميه ب.ض/810-510-523 س.ت/ 93150
المعمل الرئيسى / المقطم - الهضبه العليا - ش 9
للتدريب و عمل الابحاث / مدينة نصر - الحى العاشر - برج الزهور ب- امام مسجد الخزان

ID	Conc. ug/ml	O.D			Mean O.D	ST.E	Viability %	Toxicity %	IC50
vero	dilution	0.273	0.283	0.266	0.274	0.004933	100	0	ug
1	500	0.043	0.052	0.054	0.049667	0.003383	18.12652068	81.87347932	175.973
	250	0.06	0.054	0.074	0.062667	0.005925	22.87104623	77.12895377	
	125	0.174	0.169	0.18	0.174333	0.00318	63.62530414	36.37469586	
	62.5	0.276	0.273	0.267	0.272	0.002646	99.27007299	0.729927007	
	31.25	0.266	0.273	0.275	0.271333	0.002728	99.02676399	0.97323601	
	15.625	0.276	0.268	0.271	0.271667	0.002333	99.14841849	0.851581509	
2	500	0.046	0.051	0.047	0.048	0.001528	17.51824818	82.48175182	195.688
	250	0.086	0.09	0.097	0.091	0.003215	33.21167883	66.78832117	
	125	0.187	0.193	0.197	0.192333	0.002906	70.1946472	29.8053528	
	62.5	0.264	0.258	0.263	0.261667	0.001856	95.49878345	4.501216545	
	31.25	0.274	0.276	0.269	0.273	0.002082	99.6350365	0.364963504	
	15.625	0.277	0.276	0.269	0.274	0.002517	100	0	

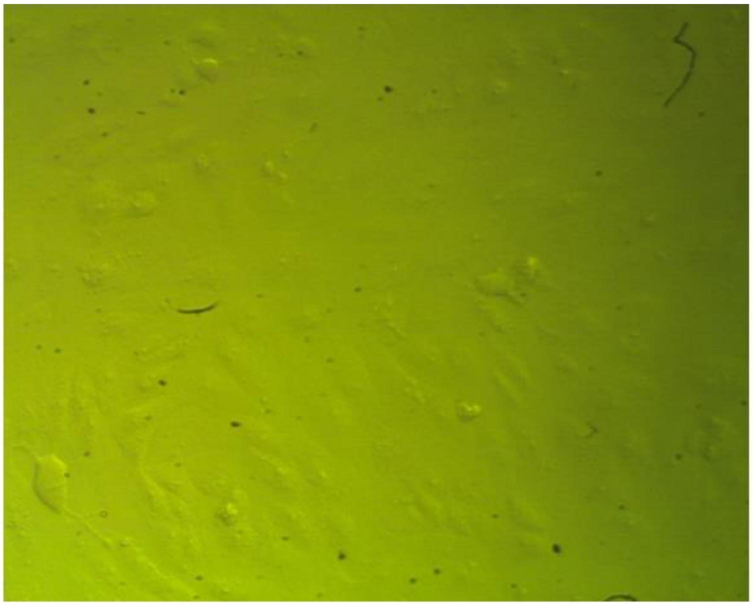

control vero cells

Organism : *Cercopithecus aethiops*
Tissue : kidney
Cell Type : epithelial
Culture Properties : adherent
Disease : normal
ATCC : CCL-81

Effect of comp. 1 on vero cells at different concentration

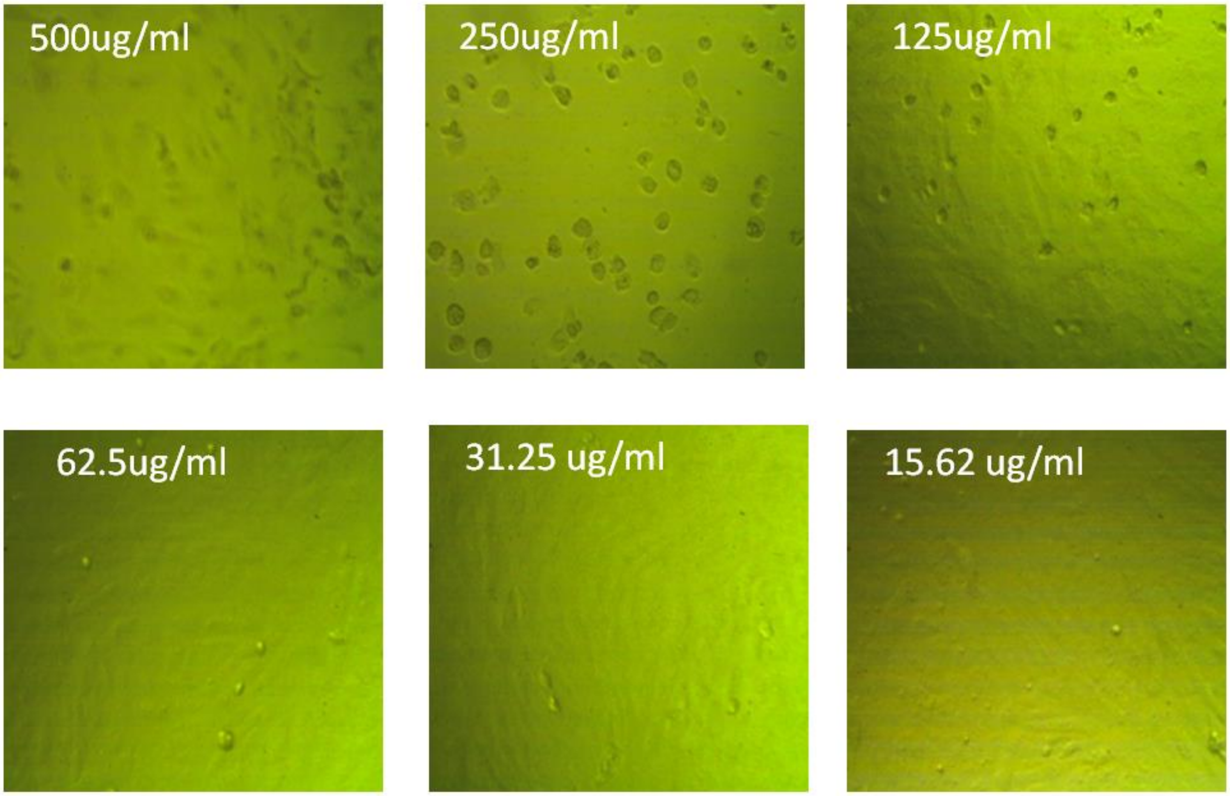

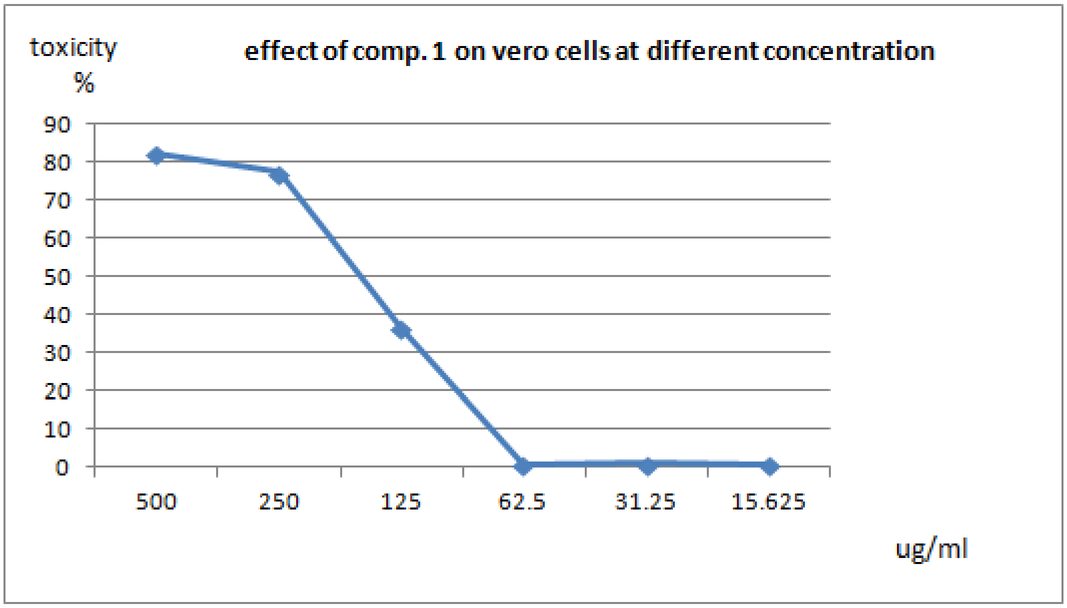

Effect of comp. 2 on vero cells at different concentration

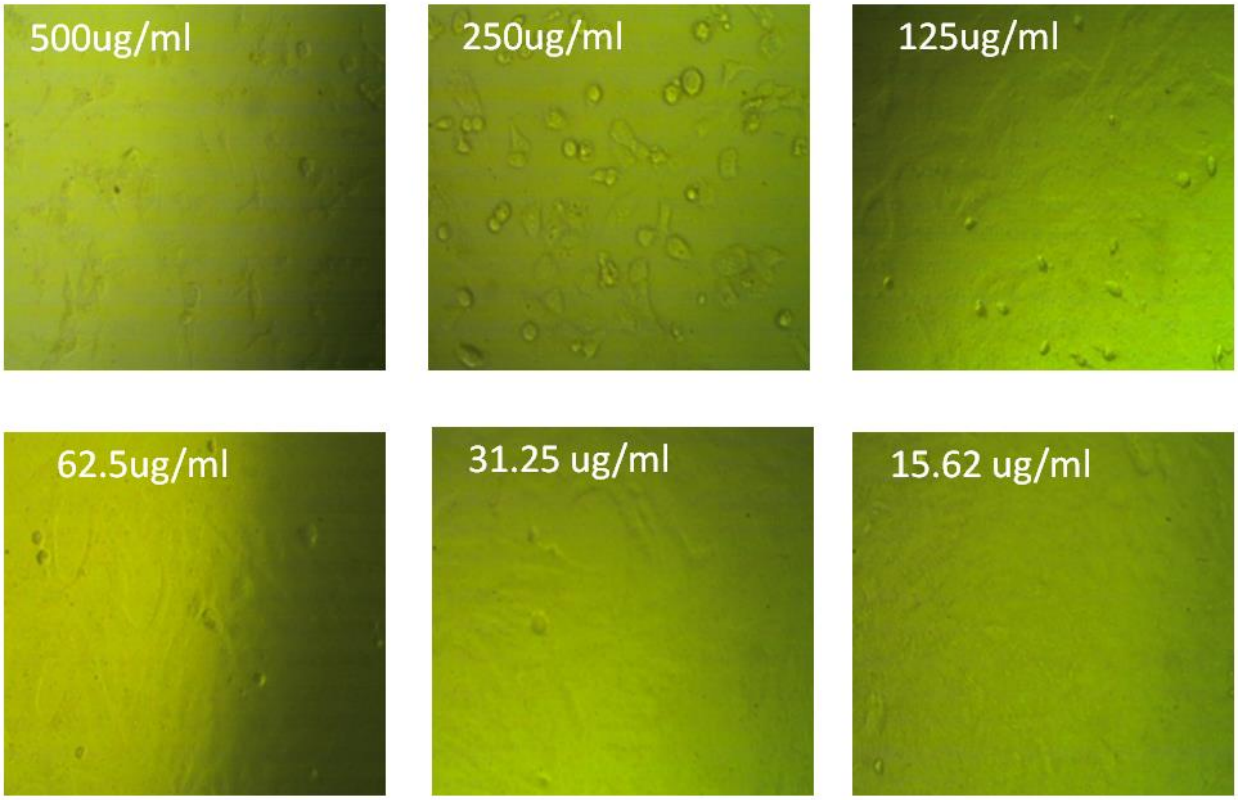

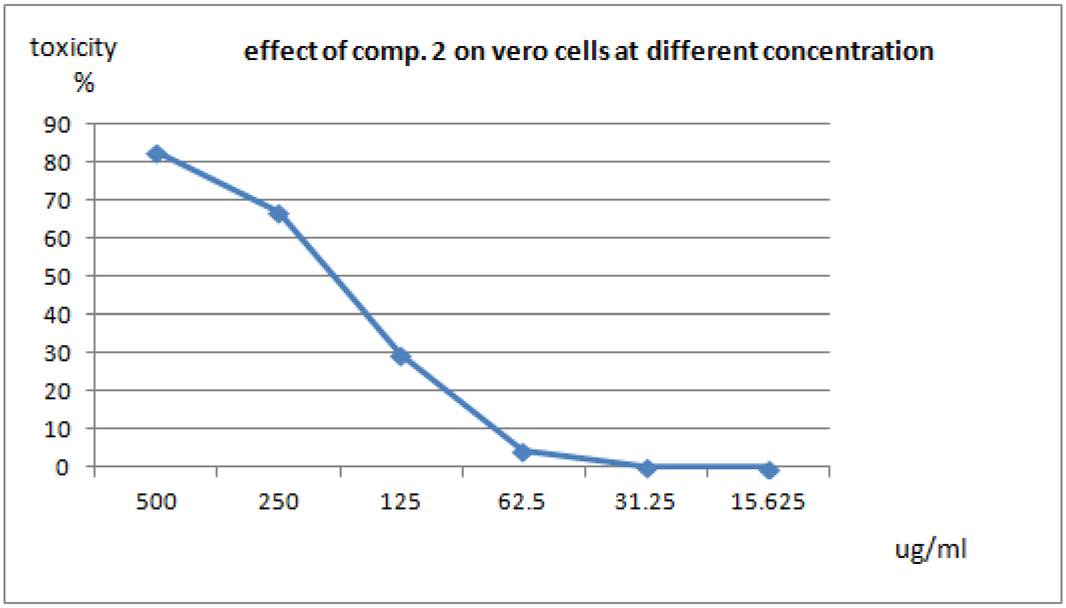

Supplement: Supplementary file 1 [file molecules-27-06724-s001.zip › T-4-019-5.pdf]
